# The architecture of cell differentiation in choanoflagellates and sponge choanocytes

**DOI:** 10.1371/journal.pbio.3000226

**Published:** 2019-04-12

**Authors:** Davis Laundon, Ben T. Larson, Kent McDonald, Nicole King, Pawel Burkhardt

**Affiliations:** 1 Marine Biological Association of the United Kingdom, The Laboratory, Citadel Hill, Plymouth, United Kingdom; 2 Plymouth University, Drake Circus, Plymouth, United Kingdom; 3 Biophysics Graduate Group, University of California, Berkeley, Berkeley, California, United States of America; 4 Electron Microscope Laboratory, University of California, Berkeley, Berkeley, California, United States of America; 5 Department of Molecular and Cell Biology, University of California, Berkeley, United States of America; 6 Howard Hughes Medical Institute, University of California, Berkeley, Berkeley, California, United States of America; 7 Sars International Centre for Molecular Marine Biology, University of Bergen, Bergen, Norway; University of Vienna, AUSTRIA

## Abstract

Although collar cells are conserved across animals and their closest relatives, the choanoflagellates, little is known about their ancestry, their subcellular architecture, or how they differentiate. The choanoflagellate *Salpingoeca rosetta* expresses genes necessary for animal development and can alternate between unicellular and multicellular states, making it a powerful model for investigating the origin of animal multicellularity and mechanisms underlying cell differentiation. To compare the subcellular architecture of solitary collar cells in *S*. *rosetta* with that of multicellular ‘rosette’ colonies and collar cells in sponges, we reconstructed entire cells in 3D through transmission electron microscopy on serial ultrathin sections. Structural analysis of our 3D reconstructions revealed important differences between single and colonial choanoflagellate cells, with colonial cells exhibiting a more amoeboid morphology consistent with higher levels of macropinocytotic activity. Comparison of multiple reconstructed rosette colonies highlighted the variable nature of cell sizes, cell–cell contact networks, and colony arrangement. Importantly, we uncovered the presence of elongated cells in some rosette colonies that likely represent a distinct and differentiated cell type, pointing toward spatial cell differentiation. Intercellular bridges within choanoflagellate colonies displayed a variety of morphologies and connected some but not all neighbouring cells. Reconstruction of sponge choanocytes revealed ultrastructural commonalities but also differences in major organelle composition in comparison to choanoflagellates. Together, our comparative reconstructions uncover the architecture of cell differentiation in choanoflagellates and sponge choanocytes and constitute an important step in reconstructing the cell biology of the last common ancestor of animals.

## Abstract 2

3D electron microscopy of choanoflagellates and sponge choanocytes reveals a remarkable variety of cell architecture and suggests that cell type differentiation may have been present in the stem lineage leading to the animals.

## Author summary

Choanoflagellates are microscopic aquatic organisms that can alternate between single-celled and multicellular states, and sequencing of their genomes has revealed that choanoflagellates are the closest single-celled relatives of animals. Moreover, choanoflagellates are a form of ‘collar cell’—a cell type crowned by an array of finger-like microvilli and a single, whip-like flagellum. This cell type is also found throughout the animal kingdom; therefore, studying the structure of the choanoflagellate collar cell can shed light on how this cell type and animal multicellularity might have evolved. We used electron microscopy to reconstruct in 3D the total subcellular composition of single-celled and multicellular choanoflagellates as well as the collar cells from a marine sponge, which represents an early-branching animal lineage. We found differences between single-celled and multicellular choanoflagellates in structures associated with cellular energetics, membrane trafficking, and cell morphology. Likewise, we describe a complex system of cell–cell connections associated with multicellular choanoflagellates. Finally, comparison of choanoflagellates and sponge collar cells revealed subcellular differences associated with feeding and cellular energetics. Taken together, this study is an important step forward in reconstructing the biology of the last common ancestor of the animals.

## Introduction

**Fig 1:**
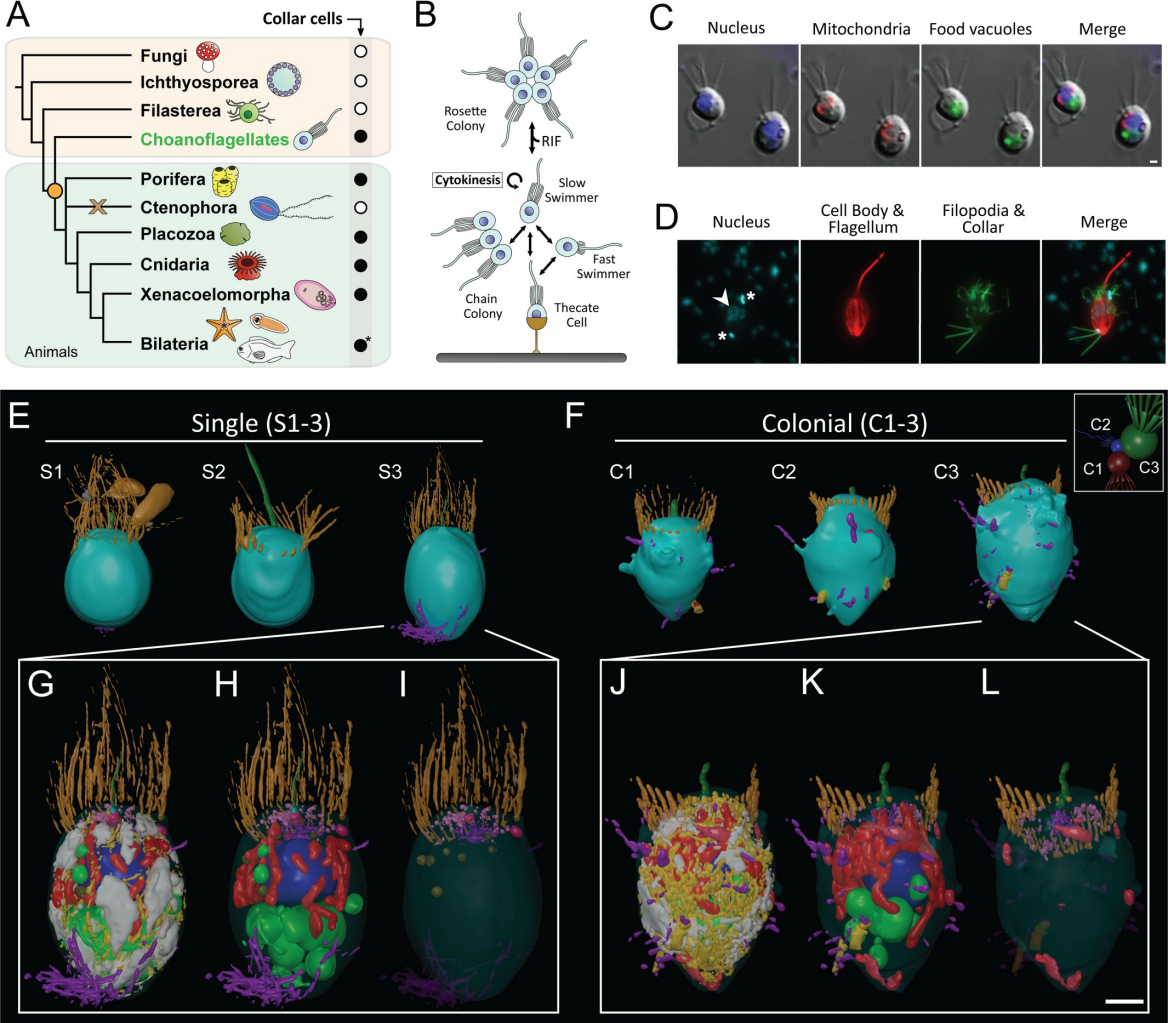
3D cellular architecture of choanoflagellates and collar cells across the Choanozoa. (A) Phylogenetic distribution of collar cells across the Choanozoa (Choanoflagellata + Metazoa [1,5]) showing the presence (black circle), absence (white circle), and putative losses (brown cross) of collar cells across lineages. The origin of collar cells is marked by the orange circle. Adapted from [1]. *Some lineages within the Bilateria have secondarily lost collar cells. (B) The choanoflagellate *S*. *rosetta* exhibits a complex life cycle, transitioning through both single and colonial collar cell types. The development of rosette colonies can be induced by RIF. Choanoflagellate colonies form through cytokinesis. (C–D) Characterisation of major organelles in *S*. *rosetta* labelled with fluorescent vital dyes (C) and by immunofluorescence (D). Arrowhead indicates nucleus of choanoflagellates cell; asterisks indicate the stained nucleoids of engulfed prey bacteria. Scale bar = 1 μm. (E–L) 3D ssTEM reconstruction of three single (S1–3) and three colonial (C1–3) *S*. *rosetta* cells (E, F). The association of the three colonial cells in context with each other are shown in the white box. The plasma membrane was made transparent (G, J), and glycogen and ER were removed to allow better visualisation of subcellular structures (H, K) and vesicle populations (I, L). Shown are apical vesicles (pink), food vacuoles (green), endocytotic vacuoles (fuschia), ER (yellow), extracellular vesicles (grey), filopodia (external, purple), flagellar basal body (light blue), flagellum (dark green), glycogen storage (white), Golgi apparatus and vesicles (purple), intercellular bridges (external, yellow; septa, red), large vesicles (brown), microvillar collar (light orange), mitochondria (red), nonflagellar basal body (dark orange), and nuclei (dark blue). Scale bar = approximately 1 μm (depending on position of structure along the z-axis). ER, endoplasmic reticulum; RIF, rosette-inducing factor; ssTEM, serial ultrathin transmission electron microscopy.

Collar cells were likely one of the first animal cell types [1–3]. Defined as apicobasally polarised cells crowned with an actin-rich microvillar collar surrounding an apical flagellum [4], they are conserved across almost all animal phyla (Fig 1A) as well as in their closest living relatives, the choanoflagellates [1]. In choanoflagellates and sponges, the undulation of the apical flagellum draws bacteria and other particulate material to the collar, where it can be phagocytosed for food. In many other animals, collar cells function as sensory epidermal cells, nephridial cells, and various inner epithelial cells [1].

Multicellularity evolved multiple times independently in eukaryotes [1,6]. Choanoflagellates are uniquely suited for investigating characteristics of the last common multicellular ancestor of animals and the origin of animal-specific innovations. Several independent phylogenomic analyses [7–9] have placed them as the closest branching lineage to the animals. It is thought that the transition from a free-swimming facultatively unicellular collar cell to one in an obligately multicellular animal condition emerged along the animal stem lineage [2]. While it has been hypothesised that the common ancestor of animals may have exhibited a complex, polymorphic life cycle [10,11], parsimony suggests that at least one of these life stages would have possessed choanoflagellate-like collar cells [1]. Investigation of the choanoflagellate cell plan therefore has the potential to shed light on the evolution of one of the most ancient animal cell types.

The colony-forming choanoflagellate *S*. *rosetta* [12] has emerged as a promising model organism to investigate the properties of the progenitor of the animals [13]. This species exhibits a complex life cycle, transitioning through both single and colonial collar cell types [12,14] (Fig 1B). The development of rosette colonies can be induced by rosette-inducing factor (RIF) (Fig 1B), which is a sulfonolipid from the bacterium *Algoriphagus machipongonensis* [15]. Most importantly, choanoflagellate colonies form by cell division, and cells within rosette colonies are held together by cytoplasmic bridges, filopodia, and extracellular matrix (ECM) [12]. Cell types of *S*. *rosetta* have been previously well investigated using molecular tools [16–18], which have revealed that choanoflagellates possess a suite of genes essential for animal multicellularity and development.

However, our structural understanding of how choanoflagellate cells like *S*. *rosetta* organise themselves into colonies—and how these compare to early-branching animal collar cells—remains unquantified relative to molecular investigations. Given the importance of cell differentiation for the origin of animals, we hypothesised that choanoflagellate colonial cells would not simply represent a cluster of single cells but would be morphologically differentiated from single cells. Our previous studies show that the proteins Flotillin and Homer colocalise in the nucleus of all single choanoflagellate cells, but not in all colonial cells providing preliminary evidence of cell differentiation within choanoflagellate rosette colonies [16]. In contrast, the nearly indistinguishable transcriptomes of single cells and colonies [17] speak against cell differentiation.

In this study, we used serial ultrathin transmission electron microsocopy (ssTEM) sectioning to reconstruct the microanatomy of unicellular and colonial *S*. *rosetta* cells to identify structural differences between collar cells in a single versus a multicellular choanoflagellate condition. To place our choanoflagellate reconstructions into the context of collar cells from an early-branching animal phylum, we reconstructed a section of a sponge choanocyte chamber from the homoscleromorph sponge *Oscarella carmela* [19] (Box 1). Our characterisation of the microanatomy of choanoflagellates and sponge choanocytes sheds light on collar cell differentiation, has implications for the origin and evolution of animal cell types, and is an important step in reconstructing the putative biology of the last common ancestor of the animals.

**Box 1. Definitions of terms used in the text**

**Amoebozoa**:

A taxonomic supergroup of eukaryotic cells capable of amoeboid locomotion.

**Homoscleromorpha**:

A taxonomic class of marine sponges displaying basement membranes between tissue layers.

**Macropinocytosis**:

A form of pinocytosis, defined as the formation of phase lucent vacuoles >0.2 μm in diameter from wave-like, plasma membrane ruffles.

**Mesohyl**:

A gelatinous matrix in sponges that occupies the space between the outer pinacoderm and inner choanoderm.

**Pseudopodium**:

An actin-rich, cytoplasm-filled cellular protrusion used for locomotion or feeding in amoeboid eukaryotic cells (plural ‘pseudopodia’).

## Results

### 3D cellular architecture of choanoflagellates

Three randomly selected single cells and three randomly selected colonial cells from a single colony were chosen for the reconstruction of entire choanoflagellate cells and subcellular structures (Fig 1, S1–S3 Figs, S1–S6 Movies). Both single and colonial *S*. *rosetta* cells exhibited a prominent, central nucleus enveloped by a mitochondrial reticulum and basal food vacuoles—as well as intracellular glycogen reserves—consistent with the coarse choanoflagellate cellular architecture reported in previous studies [20,21] (reviewed in [13,22]) (Fig 1, S1–S3 Figs, S1–S6 Movies). However, with the increased resolution of electron microscopy, we detected three morphologically distinct populations of intracellular vesicles with distinct subcellular localisations (Fig 1G, S1I–S1L Fig): 1) large vesicles (extremely electron-lucent, 226 ± 53 nm in diameter) (S1J, S1J’, and S1J” Fig); 2) Golgi-associated vesicles (electron-dense inclusions, 50 ± 10 nm in diameter) (S1I, S1I’, and S1I” Fig); and 3) apical vesicles (electron-lucent, 103 ± 21 nm in diameter) (S1K, S1K’ and S1K” Fig). Extracellular vesicles were also observed to be associated with two of the single cells (electron-lucent, 173 ± 36 nm in diameter) and appeared to bud from the microvillar membrane (S1L, S1L’ and S1L” Fig). Choanoflagellate cells subjected to fluorescent labelling were congruent with 3D ssTEM reconstructions in terms of organelle localisation (Fig 1B and 1C), providing evidence that the 3D models presented herein are biologically representative.

### Ultrastructural comparison between single and colonial choanoflagellate cells reveals surprising differences

**Fig 2:**
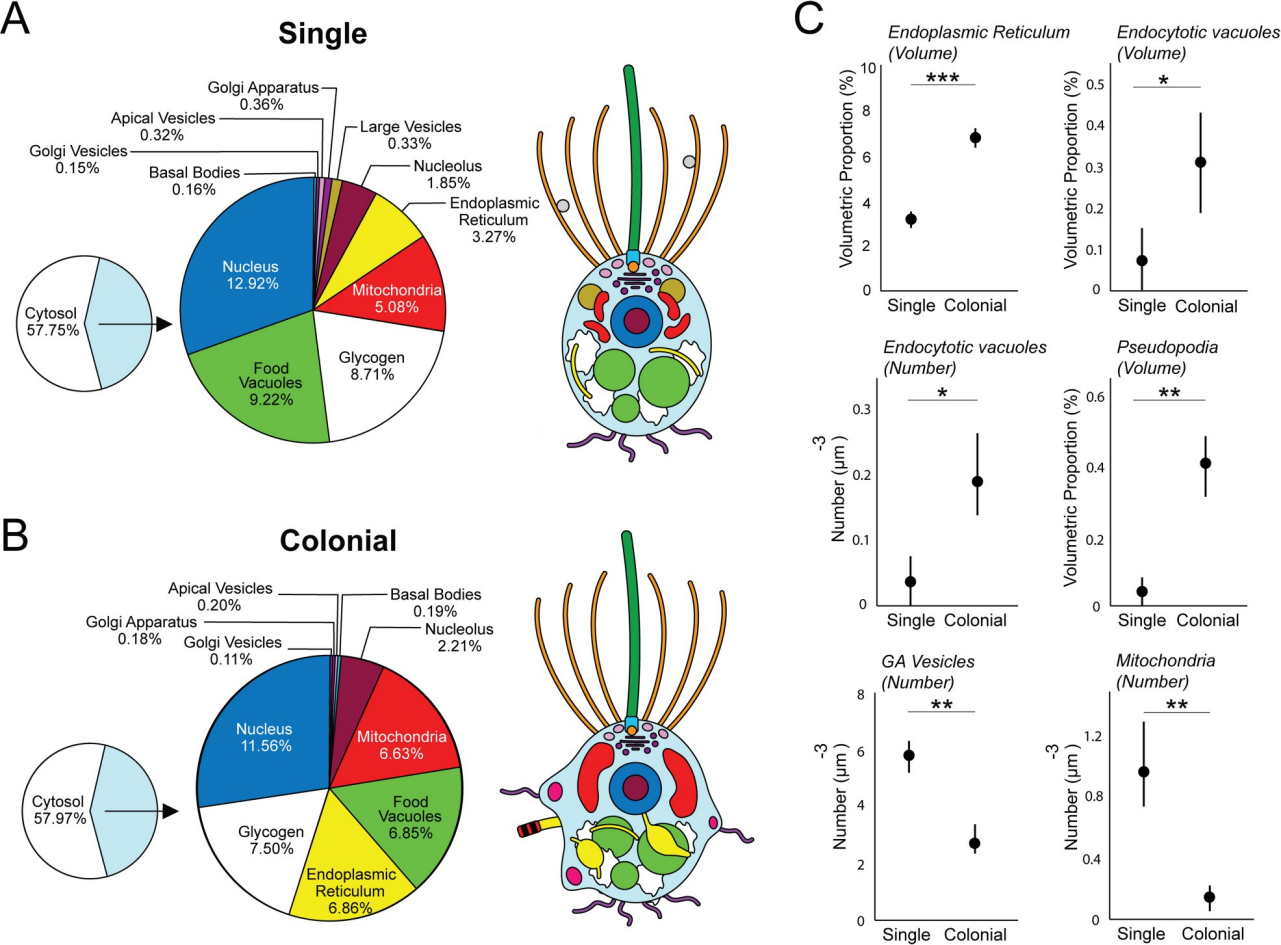
3D ssTEM reconstructions allow for volumetric and numerical comparison of high-resolution single and colonial *S*. *rosetta* cells. Shown are the mean volumetric breakdowns of three single (A) and three colonial (B) *S*. *rosetta* cells (left) and a generalised diagram of cell type ultrastructure (right). Colours are as in Fig 1. (C) Volumetric (%) (±SEM) (ER and endocytotic vacuoles) and numerical (μm^−3^) (±SEM) (endocytotic vacuoles, pseudopodia, Golgi-associated vesicles, and mitochondria) differences were found between single and colonial (*n* = 3) *S*. *rosetta* cells. *P < 0.05, **P < 0.01, ***P < 0.001. ER, endoplasmic reticulum; ssTEM, serial ultrathin transmission electron microscopy.

Our 3D ssTEM reconstructions allowed for detailed volumetric and numerical comparisons among single and colonial *S*. *rosetta* cells (Fig 2, S2 Fig, S1 and S2 Tables). Overall, the general deposition of major organelles was unchanged in both cell types (Fig 1E–1L, Fig 2A and 2B, S2A–S2C Fig). In addition, single and colonial cells devote a similar proportion of cell volume to most of their major organelles (nucleus: single cells 12.92% ± 0.58% versus colonial cells 11.56% ± 0.27%; nucleolus: 1.85% ± 0.33% versus 2.2% ± 0.22%; mitochondria: 5.08% ± 1.14% versus 6.63% ± 0.42%; food vacuoles: 9.22% ± 2.75% versus 6.85% ± 0.87%; and glycogen storage: 8.71% ± 2.36% versus 7.50% ± 1.12%) (Fig 2, S2 Fig, S1 and S2 Tables).

**Fig 3:**
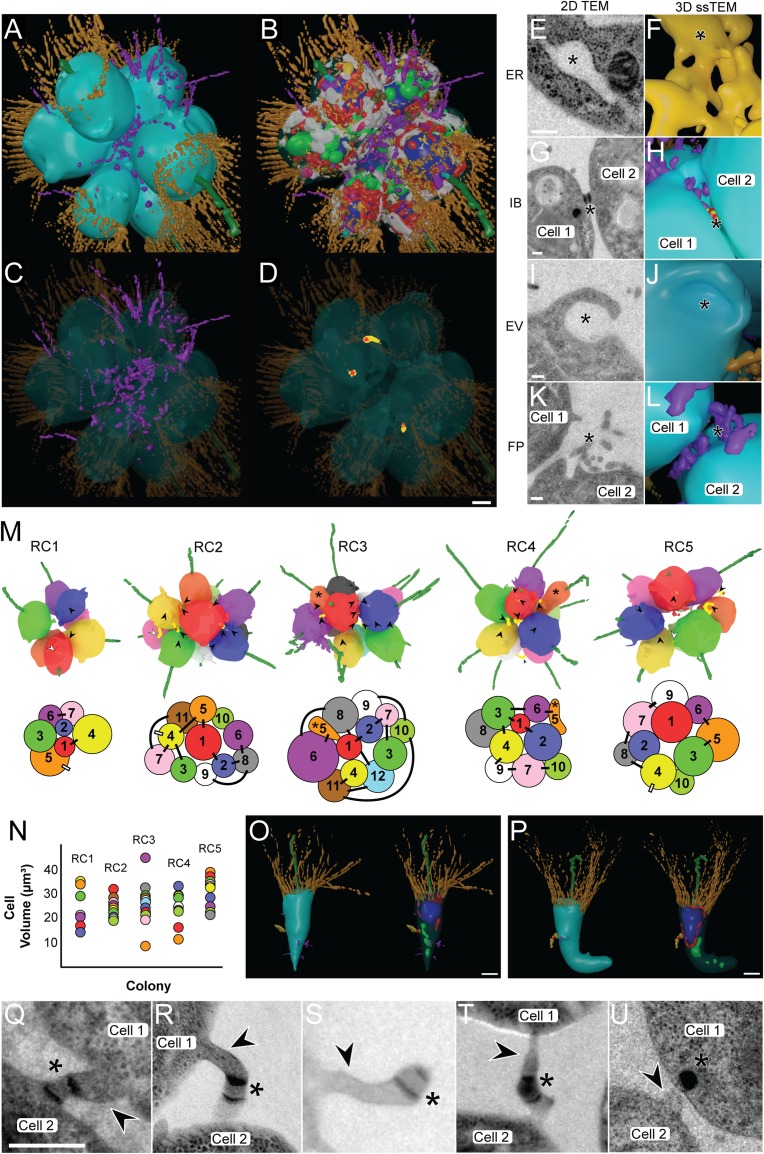
Reconstructions of complete choanoflagellate RCs places colonial cells into context and unveils ultrastructural features involved in rosette formation and a novel cell type. (A–D) 3D ssTEM reconstruction of a complete RC1. The plasma membrane was made transparent (B) to allow better visualisation of subcellular structures. Highlighted are contacting FP (C) and IBs (D). Cellular structures coloured as in Fig 1. Scale bar = approximately 1 μm. (E–L) 2D TEM and 3D ssTEM reconstructions of structures (*) differentially exhibited by colonial cells or involved in colony formation. Shown are the ER (E, F), IBs (G, H), EV (I, J), and FP (K, L). Scale bars = 200 nm. (M–P) Reconstruction of multiple *S*. *rosetta* colonies shows no strong pattern of volumetric distribution and bridge networks but reveals the presence of highly derived cell morphologies. (M) 3D ssTEM reconstructions of five complete rosettes (RC1–5) coloured by cell number (above), and 2D projections of bridge connections in 3D ssTEM reconstructions of RCs (below). Disconnected IBs marked by white arrowheads and lines. Asterisks mark the presence of highly derived cell morphologies in RC3 and RC4. Cells in RCs are numbered in order of their appearance along the z-axis. (N) Volumetric distribution of mean cell volumes (RC1–5) in RCs reveals no apparent pattern of cell distribution across the z-axis. (O, P). Two highly derived cell types, the ‘carrot cell’ (O) from RC3 and the ‘chili cell’ (P) from RC4, were identified in RCs. Colours as in Fig 1. Scalebar = approximately 1 μm. (Q–U) IBs in colonial *S*. *rosetta* exhibit a high diversity of morphologies, suggestive of disconnection. In addition to prior descriptions of IBs (arrowheads) and electron-dense septa (asterisks), bridges in colonial *S*. *rosetta* often display an asymmetrically distributed septum (Q), protracted and elongated morphology (R), disconnection from one of the contiguous cells (S), evidence of abscission (T), and putative inheritance of the septum (U). Scale bar = 200 nm. ER, endoplasmic reticulum; EV, endocytotic vacuoles; FP, filopodia; IB, intercellular bridge; RC, rosette colony; ssTEM, serial ultrathin transmission electron microscopy.

We did, however, uncover some interesting ultrastructural differences between single and colonial cells (Fig 2C). Colonial cells devoted a higher proportion of cell volume to endoplasmic reticulum (ER) (single: 3.27% ± 0.35% versus colonial: 6.86% ± 0.39%). This contrast was coupled to a differential ER morphology across cell types. The ER of colonial cells frequently displayed wide, flat sheets (Fig 3E), which were not observed in the reconstructed single cells. Single cells exhibited a higher number of Golgi-associated vesicles (single: 166.3 ± 32.7 versus colonial: 72.3 ± 26.5) and individual mitochondria than colonial cells (single: 25.3 ± 5.8 versus colonial: 4.3 ± 4.2) (Fig 2C, S2 Table) despite lacking volumetric differences between cell types.

Finally, we found that colonial cells are characterised by a more amoeboid morphology than single cells (Fig 3A). Colonial cells exhibited a higher relative proportion of endocytotic vacuoles by volume (single: 0.07 ± 0.07 versus colonial: 0.32 ± 0.12)—a phenomenon coupled to a higher overall number of endocytotic vacuoles (single: 1 ± 1 versus colonial: 5 ± 2) and pseudopodial projections per cell (single: 1 ± 1 versus colonial 8 ± 2) (Fig 2C, S1 and S2 Tables). Many of the pseudopodial projections and endocytotic vacuoles bore the morphology of lamellipod ruffles and macropinosomes (Fig 3A), suggesting that colonial cells are typified by high macropinocytotic activity.

### Reconstruction of multiple rosettes reveals colony-wide cell arrangement, different cell shapes, and complete cell–cell contact networks

While high-magnification 3D ssTEM enabled the high-resolution reconstruction of individual colonial cells, their context and interactions with neighbouring cells were lost. To address this, we reconstructed the subcellular structures of a seven-cell rosette colony (complete rosette, RC1) from 80-nm sections taken at lower magnification (Fig 3A–3D, S7 Movie) as well as the gross morphology of four larger rosettes (RC2–5) from 150-nm sections to provide a more representative survey (Fig 3E–3P). We found that individual cells in rosette colonies vary widely in volume (Fig 3M and 3N), although no pattern was detected in the volumetric cellular arrangement along the rosette z-axis (Fig 3M). In addition, mean cell size was comparable among different rosettes, including those that contained different numbers of cells (S4B Fig). However, we did find a positive correlation between cell number and the number of intercellular bridges per cell across rosette colonies (S4B Fig).

Importantly, we uncovered the presence of unusually shaped cells in two of the five *S*. *rosetta* rosette colonies (carrot-shaped cell 5 in RC3 and chili-shaped cell 5 in RC4, both labelled orange with an asterisk) (Fig 3M). These unusual cells were both found at the same location along the rosette z-axis, exhibited an elongated morphology distinct from other colonial cells (Fig 3O and 3P and S8 and S9 Movies), and were small in volume. Cells 5 from RC3 and RC4 were 9.87 μm^3^ and 13.35 μm^3^, respectively (Fig 3N)—the mean volume of the cells in RC3 and RC4 was 27.38 μm^3^ and 27.25 μm^3^, respectively (Fig 3N). While each of these unusual cells possessed a flagellum, a collar, connections to neighbouring cells via intercellular bridges, and had a similar proportion of cell volume dedicated to most of their major organelles as observed in other colonial cells, these cells devoted a larger volumetric percentage of the cell body to the nucleus (29.8% and 30.78%, respectively, versus the mean colonial proportion of 13.76% ± 0.49%).

Our 3D ssTEM reconstructions of rosette colonies also revealed the distribution of intercellular bridges and the connections formed between individual cells (Fig 3M). We found intercellular bridges in all analysed rosette colonies (RC1–5), totalling 36 bridges. There was no detectable pattern regarding bridge networking across rosette colonies. Bridges were distributed from the cell equator to either of the poles along the cellular z-axis, and the average bridge was 0.75 ± 0.38 μm in length (S4C Fig). Prior studies [12,17] of *S*. *rosetta* bridges suggested that bridges are typically short (0.15 μm), connecting two adjacent cells and containing parallel plates of electron-dense material. In contrast, the bridges detected in this study exhibited striking morphological diversity (Fig 3M, 3Q–3U), with lengths ranging from 0.21–1.72 μm. The majority of bridges consisted of a protracted cytoplasmic connection between two cells, and in many cases, the septum was localised asymmetrically along the bridge (S4C Fig). Most surprisingly, some bridges were not connected to any neighbouring cells at all, but rather the septum was situated on the end of a thin, elongated cellular protrusion (Fig 3S). In addition, we observed asymmetric bridge width and degraded electron-dense structures proximal to bridge remnants being incorporated into the cell body of a contiguous cell (Fig 3T and 3U). These data suggest that intercellular bridges could be disconnected from neighbouring cells and that the electron-dense septum may be inherited.

### Comparison of 3D cellular architecture between choanoflagellates and sponge choanocytes

**Fig 4:**
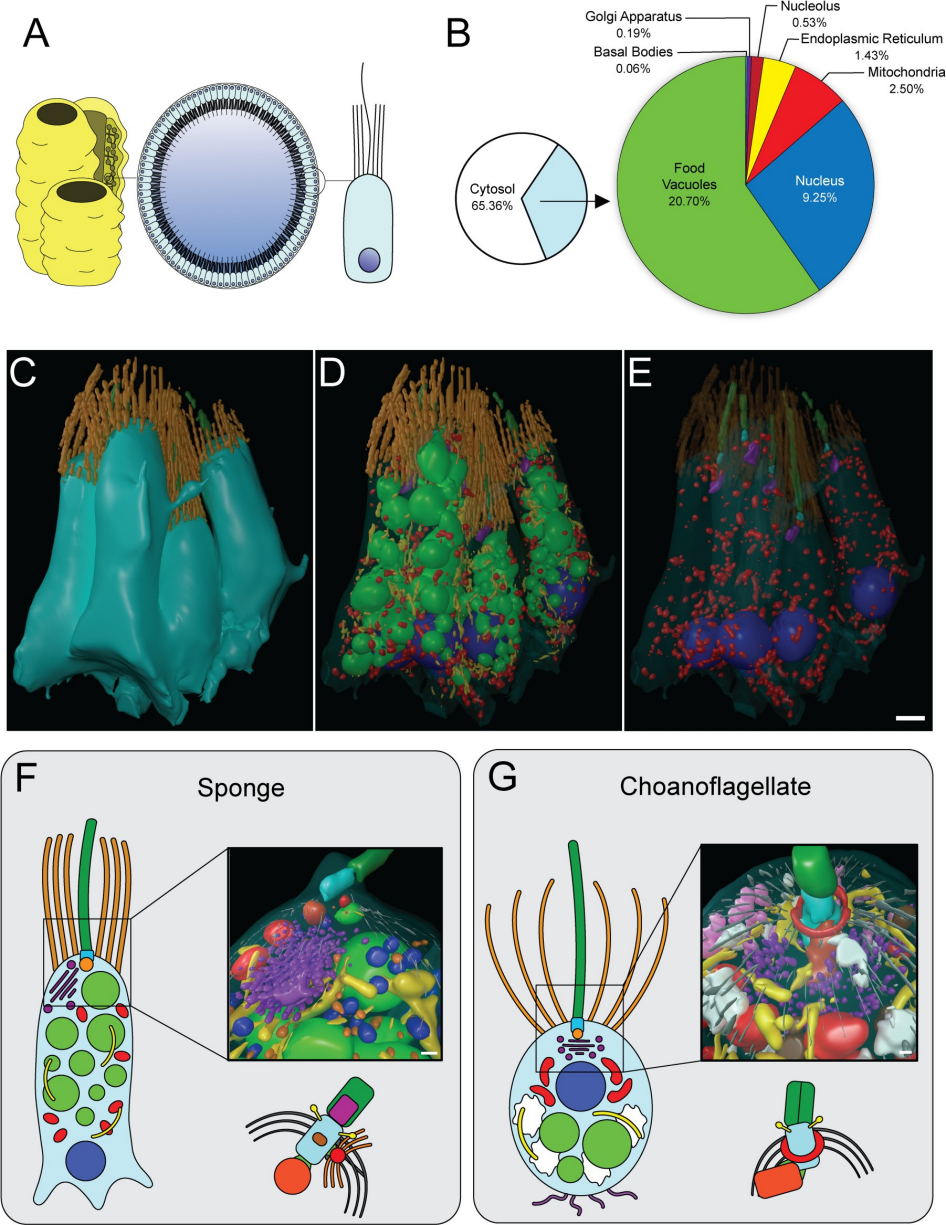
3D cellular architecture of sponge choanocytes. (A) Choanocytes line interconnected chambers in members of the Porifera and serve as feeding cells. (B) Mean volumetric breakdown of five sponge choanocytes. Colours are as in Fig 1. (C–E) 3D ssTEM of a section of choanocyte chamber containing five complete cells (B). The plasma membrane was rendered transparent (D), and food vacuoles and ER were removed to allow better visualisation of subcellular structures (E). Colours are as in Fig 1. Scale bar = approximately 1 μm. (F–G) Reconstruction and comparison of the sponge choanocyte (F) and choanoflagellate (G) apical poles shows distinct differences between the two cell types. Shown in the choanocyte reconstruction are the basal foot (red, associated with basal body), food vacuole (light green), ER (yellow), flagellar basal body (light blue), flagellum (dark green), Golgi apparatus and Golgi-associated vesicles (purple), microtubules (grey), mitochondria (red), nonflagellar basal body (dark orange), Type 1 vesicles (light orange), and Type 2 vesicles. Shown in the choanoflagellate reconstruction are the apical vesicles (pink), food vacuole (light green), ER (yellow), flagellar basal body (light blue), flagellum (dark green), Golgi apparatus and Golgi-associated vesicles (purple), glycogen (white), large vesicles (brown), microtubules (grey), microtubular ring (red), and nonflagellar basal body (dark orange). Scale bars = 200 nm. Diagrams of the choanocyte fine kinetid (F) and choanoflagellate fine kinetid (G) structure highlight the distinct differences. ER, endoplasmic reticulum; ssTEM, serial ultrathin transmission electron microscopy.

Both choanoflagellates and sponge collar cells influence local hydrodynamics by beating their single flagellum to draw in bacteria that are captured by the apical collar complex [23], however sponge choanocytes are part of an obligately multicellular organism (Fig 4A). Sponge choanocytes therefore provide an excellent representative of an early-branching animal collar cell against which to compare choanoflagellate cell architectures. Our 3D ssTEM reconstructions allowed for the reconstruction of five choanocytes and for the volumetric and numerical comparison of choanocyte and choanoflagellate subcellular structures (Fig 4B–4E, S5 and S6 Figs, S10 Movie). We detected little ultrastructural variability between the five choanocytes (S5 Fig, S3 and S4 Tables). All five cells exhibited a prominent basal nucleus, small and unreticulated mitochondria, food vacuoles scattered around the entire cell, and an apical Golgi apparatus (Fig 4B–4D, S5 and S6 Figs)—consistent with the coarse choanocyte cellular architecture reported in previous studies [23,24] (reviewed in [1,25]).

Furthermore, our data showed many ultrastructural commonalities between sponge choanocytes and choanoflagellates. For example, the number of microvilli that surround the apical flagellum in single and colonial choanoflagellates is comparable to the number of microvilli in sponge choanocytes (single: 32 ± 2 versus colonial: 35.3 ± 4.9 versus choanocytes: 30.6 ± 4.1) (S6A Fig). We also found that the number of food vacuoles and the number and volumetric proportion of the Golgi apparatus are similar in all three cell types (S6A Fig). Although sponge choanocytes did not appear to exhibit the same macropinocytotic activity as colonial choanoflagellates throughout the cell (some micropinocytotic inclusions are present toward the cell apex [S6D and S6E Fig]), basal sections of choanocytes were heavily amoeboid (S6B and S6C Fig). These amoeboid protrusions may not only be for mechanical anchorage into the mesohyl but may play a role in phagocytosis, as we observed bacteria in the mesohyl to be engulfed by basal pseudopodia (S6F and S6G Fig). Thus, both choanocytes and colonial choanoflagellates are typified by high-amoeboid cell activity.

We also observed some ultrastructural differences between choanocytes and choanoflagellates. In contrast with cells from choanoflagellate rosettes, sponge choanocytes lack filopodia and intercellular bridges. Choanocytes also do not possess glycogen reserves and devote significantly less of their cell volume (9.25% ± 0.39%) than choanoflagellates (single: 12.92% ± 0.58% and colonial: 11.56% ± 0.27%) to the nucleus and less to mitochondria (2.5% ± 0.3% versus single: 5.08% ± 1.14% and colonial: 6.63% ± 0.42%) (S6A Fig). However, choanocytes devote significantly more of their volume to food vacuoles (20.7% ± 1.01%) than choanoflagellates (single: 9.22% ± 2.75% and colonial: 6.85% ± 0.87%) (Fig 4E). High-resolution reconstructions of the choanocyte and choanoflagellate apical pole (Fig 4F and 4G, S11 and S12 Movies) showed differences in terms of vesicle type and localisation, Golgi positioning, and collar arrangement (conical in choanoflagellates while cylindrical in choanocytes, as previously noted [23]). The flagellar basal body has previously been meticulously characterised in both choanocytes and choanoflagellates, and some differences have been reported between the two by other authors [26–31]. These findings are reiterated by our reconstructions and observations (Fig 4F and 4G).

## Discussion

Our comparison between single and colonial choanoflagellate cells provides new insights into ultrastructural commonalities and differences associated with the conversion of solitary to colonial cells. Our study also revealed morphologically distinct populations of vesicles in choanoflagellates. Golgi-associated vesicles (S1I, S1I’ and S1I” Fig), due to their tight association with the Golgi apparatus, likely represent standard Golgi-trafficking vesicles carrying cargo between the different Golgi cisternae [4]. Apical vesicles (S1K, S1K’ and S1K” Fig), due to their close proximity to the plasma membrane, are probably secretory vesicles involved in exocytosis of ECM material [4], which bud off the trans-Golgi network and fuse with the plasma membrane. The localisation of neurosecretory soluble N-ethylmaleimide-sensitive-factor attachment receptor (SNARE) proteins to the apical pole of the choanoflagellate *Monosiga brevicollis* supports this hypothesis [32,33]. The large vesicles (S1J, S1J’ and S1J” Fig) may not represent true vesicles but rather nascent food vacuoles, congruent with what is already known about phagocytosis in choanoflagellates [34]. The finding of extracellular vesicles (S1L, S1L’ and S1L” Fig) associated with the *S*. *rosetta* microvillar collar is, to our knowledge, a novel finding in choanoflagellates. Extracellular vesicles in animal cells play diverse roles in cell physiology, such as antigen presentation (reviewed by [35]), morphogenesis [36,37], and disseminating pathogenic proteins [38,39]. Association of extracellular vesicles with apical microvilli, as reported here in *S*. *rosetta*, bears a striking similarity to animal enterocytes [40]. Extracellular vesicles released from enterocyte microvilli are enriched in intestinal alkaline phosphatase and are thought to be antibacterial in nature [40,41]. It is therefore conceivable that choanoflagellate extracellular vesicles too contain hydrolytic enzymes to catalyse the degradation of bacteria in the collar—the site of prey capture [34].

Moreover, our findings reveal that colonial cells likely represent distinct and differentiated cell types relative to single cells. The ultrastructural differences between single and colonial cells in ER, Golgi-associated vesicles, and the amoeboid and pinocytotic nature of colonial cells hint toward a demand on endomembrane reorganisation and intracellular trafficking (one possibility could be the increased uptake of RIF-1 to keep colonies intact). ER and mitochondrial morphology change dynamically, and stark changes have been observed in other eukaryotic cells due to changes in cell cycle [42] and cytoskeletal activity [43,44]. Mitochondria and the ER also show an intimate association [45], and the contrast in the number of individual mitochondria in different choanoflagellate cells was particularly striking. The reduced numbers of mitochondria in colonial cells indicate a lower energy consumption than in single cells. The demand on energy in single cells, which have to swim to find new food sources, may well be higher than in colonial cells, which do not have to swim, as they tumble to stay in food-rich environments. In animal cell types, fusion/fission dynamics have been previously associated with cellular stress [46] and substrate availability [47], but it is of most interest for choanoflagellates in the context of aerobic metabolism. For example, the fresh water choanoflagellate *Desmarella moniliformis* exhibits a shift in mitochondrial profile prior to encystment and metabolic dormancy [48], and choanoflagellates have been uncovered from hypoxic waters [49]. The role of oxygen in the origin and evolution of the animals has long been discussed [50] and is currently met with controversy [51,52]. Coupled to a previous report of positive aerotaxis in *S*. *rosetta* rosette colonies [53], our finding emphasises the need to better understand variation in aerobic metabolism between single and colonial choanoflagellates.

Particularly surprising was the finding of extensive macropinocytotic activity in colonial cells. Macropinocytosis—defined by the formation of phase-lucent vacuoles >0.2 μm in diameter from wave-like, plasma membrane ruffles [54]—is conserved from the Amoebozoa [55] to animal cell types [56]. It is parsimonious to infer that the macropinocytotic activity of *S*. *rosetta* colonial cells represents a trophic adaptation considering that previous biophysical studies have reported more favourable feeding hydrodynamics in rosette colonies [57], although a more recent study does not confirm these findings [53]. Even in macropinosomes with no observable cargo, dissolved proteins [58] and ATP [56] from extracellular fluid have been previously reported to be metabolically exploited by animal macropinocytotic cell types. However, this nonselectivity, coupled with the large volume of engulfed fluid, makes macropinocytosis an efficient cellular process to sample the extracellular milieu. It is therefore tempting to speculate that macropinocytosis may also play a role in detecting environmental chemical signals in colonial *S*. *rosetta* cells.

The reconstruction of multiple choanoflagellate rosette colonies reveals the asymmetric and disconnected morphology of intercellular bridges and provides important clues to choanoflagellate colony formation and potentially the evolution of animal multicellularity. Bridges displaying electron-dense septa reminiscent of those found in *S*. *rosetta* have been previously identified in other colony-forming choanoflagellate species [59], and it has been hypothesised that these structures represent stable channels for intercellular communication [17]. Our data suggest that bridges can be disconnected and that the electron-dense septum may be asymmetrically inherited. In this way, choanoflagellate bridges may resemble the mitotic midbody in animal cells [60]. Relatively recent molecular studies have suggested that inheritance of the mitotic midbody may be associated with diverse developmental roles [61–63] in the recipient cell. While homology between the electron-dense septum in choanoflagellates and metazoan midbodies cannot be determined from these data, asymmetric inheritance of this structure could play an analogous role in the development of colonial cells. It may still be that *S*. *rosetta* bridges play a role in cell–cell communication, albeit transiently. However, the exit of colonial cells from the rosette (as previously reported [12]) must involve bridge disconnection, and a proper understanding of the fate of the septum could augment our understanding of choanoflagellate cell differentiation and destiny in colony development.

The discovery of the highly derived ‘carrot’ and ‘chili’ cell types was not expected (Fig 3O and 3P). The morphological similarity, the enlarged nuclear volume, and the position on the colony z-axis shared between the two cells suggests that they represent a distinct *S*. *rosetta* cell type in rosette colonies. These data hint that cell differentiation within colonies may be more complex than previously realised and provide potential evidence for division of labour in choanoflagellate colonies. Previously proposed models of animal evolution via a colonial intermediate place emphasis on cellular differentiation and division of labour as key innovations toward obligate animal multicellularity [2,64]. We cannot exclude the possibility that the carrot- and chili-shaped cells are results of cells preparing to divide or cells shortly after cell division, but we think this is highly unlikely. There is no precedence for it in the literature, and our own live-cell observations of choanoflagellate cell divisions do not support this either. ‘Chili’ and ‘carrot’ cells in choanoflagellate colonies might be either caused by programmatic cellular differentiation or stochastic developmental noise. Cells in rosettes, which have different numbers of intracellular bridges and adjacent cells, may sense (through macropinocytosis) and respond (through apical vesicles) to the local environment of cells, thus making stochastic, cell-autonomous differentiation more likely than deterministic cell differentiation. We cannot rule out either of these causes at the moment but believe further research into the cell biology of these putative novel cell types is desperately needed, and single-cell transcriptomic data and live-cell imaging of choanoflagellate rosette development could shed more light on cell type variation in rosette colonies.

The 3D cellular architecture of sponge choanocytes allowed for the detailed comparison of their architecture with choanoflagellates. Although we observed many ultrastructural similarities between choanoflagellates and sponge choanocytes, there were noteworthy differences in terms of food vacuole, mitochondria, and glycogen composition. This is likely due to the different physiological niches occupied by the two cell types. As free-living protists, choanoflagellates must maintain energetically costly motility and may devote a larger proportion of their cytoplasm to mitochondrial reticula and glycogen stores at the expense of food vacuoles. Choanocytes are but one cell type in a sessile multicellular organism that exhibits cellular differentiation and strict division of labour. As such, choanocytes represent a specialised feeding cell type (which devotes a significantly higher amount of its cell volume to food vacuoles) that operates a vastly different physiology to the independent ancestral collar cell. These ultrastructural differences are good identifying features marking the differential biology of generalist versus specialist collar cells.

Recently, morphological and functional differences between choanocytes and choanoflagellates have been taken as evidence that collar cells have evolved by convergent evolution for feeding on bacteria [23,65]. Evolution is expected to lead to differences among homologous structures (an excellent example are vertebrate limbs that are all very different—some are wings, some are legs, some are fins but are still homologous) and thus it is not surprising to observe (ultra)structural differences between choanoflagellates and choanocytes.

### Concluding remarks

While we recognise the limitations of our findings due to the morphological descriptive nature of this study and the small sample size, the comparative 3D reconstruction of collar cells from two different phyla, choanoflagellates and sponges, allowed for an unbiased view of their cellular architecture and for the reconstruction of key properties of the enigmatic ancestral collar cell. Our data reveal distinct ultrastructural features in single and colonial choanoflagellates and demonstrate that cells within rosette colonies vary significantly in their cell size and shape. The newly identified ‘carrot’ and ‘chili’ cells reveal that cells within choanoflagellate colonies do not simply consist of an assemblage of equivalent single cells, but some may represent a distinctly differentiated cell type displaying ultrastructural modifications. Likewise, our data suggest that sponge choanocytes are not simply an incremental variation on the choanoflagellate cell plan but are specialised feeding cells, as indicated by their high volumetric proportion of food vacuoles. Together, our data show a remarkable variety of collar cell architecture and suggest cell type differentiation may have been present in the stem lineage leading to the animals.

## Materials and methods

### Cell culture

Colony-free *S*. *rosetta* cultures (ATCC 50818) were grown with coisolated prey bacteria in 0.22 μm filtered choanoflagellate growth medium [66] diluted at a ratio of 1:4 with autoclaved seawater. Cultures were maintained at 18°C and split 1.5:10 once a week. Colony-enriched *S*. *rosetta* cultures (PX1) were likewise maintained but monoxenically cultured with the prey bacterium *A*. *machipongonensis* [67] to induce rosette formation.

### Fluorescent labelling of organelles

To support the annotation of organelles from ssTEM sections, the microanatomy of *S*. *rosetta* cells was chemically characterised by fluorescent vital staining. Cells were pelleted by gentle centrifugation (500x g for 10 min at 4°C) in a Heraeus Megafuge 40R (ThermoFisher Scientific) and resuspended in a small volume of culture medium. Concentrated cell suspension (500 μl) was applied to glass-bottom dishes, coated with poly-L-lysine solution (P8920, Sigma-Aldrich), and left for 10–30 min until cells were sufficiently adhered. PX1 cultures were concentrated into 100 μl of culture medium to promote the adherence of rosette colonies.

Adhered cells were incubated in 500 μl of fluorescent vital dye diluted in 0.22 μm filtered seawater. Cells were incubated with 4.9 μM Hoechst 33342 Dye for 30 min (to label nuclei), 1 μM LysoTracker Yellow HCK-123 for 1.5 h (to label food vacuoles), and 250 nM MitoTracker Red CM-H2Xros for 30 min (to label mitochondria). All vital dyes were from ThermoFisher Scientific (H3570, L12491, T35356, and M7513, respectively). Fluorescent-DIC microscopy was conducted under a 100x oil-immersion objective lens using a Leica DMi8 epifluorescent microscope (Leica, Germany). Vital dyes were viewed by excitation at 395 nm and emission at 435–485 nm (Hoechst 33342 Dye), 470 nm and emission at 500–550 nm (LysoTracker Yellow HCK-123 and FM 1–43 Dye), and 575 nm and 575–615 nm (MitoTracker Red CM-H2Xros). Micrographs were recorded with an ORCA-Flash4.0 digital camera (Hamamatsu Photonics, Japan). All cells were imaged live. No-dye controls using only the dye solvent dimethyl sulfoxide (DMSO) (D4540, Sigma-Aldrich) were run for each wavelength to identify and control for levels of background fluorescence. Chemical fixation during vital staining and TEM sectioning was avoided where possible in this study to reduce fixation artefacts.

To visualise cell bodies, flagella, filopodia, and collar-adherent cells were fixed for 5 min with 1 ml 6% acetone and for 15 min with 1 ml 4% formaldehyde. Acetone and formaldehyde were diluted in artificial seawater, pH 8.0. Cells were washed gently four times with 1 ml washing buffer (100 mM PIPES at pH 6.9, 1 mM EGTA, and 0.1 mM MgSO_4_) and incubated for 30 min in 1 ml blocking buffer (washing buffer with 1% BSA and 0.3% Triton X-100). Cells were incubated with primary antibodies against tubulin (E7, 1:400; Developmental Studies Hybridoma Bank), diluted in 0.15 ml blocking buffer for 1 h, washed four times with 1 ml of blocking buffer, and incubated for 1 h in the dark with fluorescent secondary antibodies (1:100 in blocking buffer, Alexa Fluor 488 goat anti-mouse). Coverslips were washed three times with washing buffer, incubated with Alexa Fluor 568 Phalloidin for 15 min, and washed again three times with washing buffer. Coverslips were mounted onto slides with Fluorescent Mounting Media (4 ml; Prolong Gold Antifade with DAPI, Invitrogen). Images were taken with a 100x oil-immersion objective on a Leica DMI6000 B inverted compound microscope and Leica DFC350 FX camera. Images presented as z-stack maximum intensity projections.

### Electron microscopy

#### High-pressure freezing

Cultured *S*. *rosettta* single and colonial cells were concentrated by gentle centrifugation (500x g for 10 min), resuspended in 20% BSA (Bovine Serum Albumin, Sigma) made up in artificial seawater medium, and concentrated again. Most of the supernatant was removed and the concentrated cells transferred to high-pressure freezing planchettes varying in depth between 50 and 200 μm (Wohlwend Engineering). For sponges, tiny pieces of *O*. *carmela* were excised and mixed with 20% BSA made up in seawater before transferring to 200-μm deep high-pressure freezing planchettes. Freezing of both the choanoflagellate and sponge samples was done in a Bal-Tec HPM-010 high-pressure freezer (Bal-Tec AG).

#### Freeze substitution

High-pressure frozen cells stored in liquid nitrogen were transferred to cryovials containing 1.5 ml of fixative consisting of 1% osmium tetroxide plus 0.1% uranyl acetate in acetone at liquid nitrogen temperature (−195°C) and processed for freeze substitution according to the method of McDonald and Webb [68,69]. Briefly, the cryovials containing fixative and cells were transferred to a cooled metal block at −195°C—the cold block was put into an insulated container such that the vials were horizontally oriented—and shaken on an orbital shaker operating at 125 rpm. After 3 h, the block/cells had warmed to 20°C and were ready for resin infiltration.

#### Resin infiltration and embedding

Resin infiltration was accomplished according to the method of McDonald [69]. Briefly, cells were rinsed three times in pure acetone and infiltrated with Epon-Araldite resin in increasing increments of 25% over 30 min plus three changes of pure resin at 10 min each. Cells were removed from the planchettes at the beginning of the infiltration series and spun down at 6,000x g for 1 min between solution changes. The cells in pure resin were placed in between two PTFE-coated microscope slides and polymerised over 2 h in an oven set to 100°C.

#### Serial sectioning

Cells/tissues were cut out from the thin layer of polymerised resin and remounted on blank resin blocks for sectioning. Serial sections of varying thicknesses between 70–150 nm were cut on a Reichert-Jung Ultracut E microtome picked up on 1 x 2-mm slot grids covered with a 0.6% Formvar film. Sections were poststained with 1% aqueous uranyl acetate for 7 min and lead citrate [70] for 4 min.

#### Imaging

Images of cells on serial sections were taken on an FEI Tecnai 12 electron camera.

### 3D reconstruction and analysis

ssTEM sections were imported as z-stacks into the Fiji [71] plugin TrakEM2 [72] and automatically aligned using default parameters, except for increasing steps per octave scale to 5 and reducing maximal alignment error to 50 px. Alignments were manually curated and adjusted if deemed unsatisfactory. Organelles and subcellular compartments were manually segmented and 3D reconstructed by automatically merging traced features along the z-axis. Meshes were then preliminarily smoothed in TrakEM2 and exported into the open-source 3D software Blender 2.77 [73]. Heavy smoothing of the cell body in TrakEM2 sacrifices fine structures associated with cellular projections or does not remove all distinct z-layers, which exist as reconstruction artefacts. Therefore, cell bodies were manually smoothed using the F Smooth Sculpt Tool in Blender of final distinct z-layers for presentation purposes only (S3 Fig). All organelles were subjected to the same smoothing parameters across individual cells. All analysis was conducted using unsmoothed, unprocessed meshes. Organelle volumes were automatically quantified by the TrakEM2 software and enumerated in Blender 2.77 by separating meshes in their total loose parts.

The microvillar collar and flagellum were excluded from volumetric analysis, as their total, representative length could not be imaged at this magnification. Cytosolic volume was calculated by subtracting total organelle volume from cell body volume and is inclusive of cytosol, ribosomes, and unresolved smaller structures excluded from 3D reconstruction. Endocytotic vacuoles were distinguished from food vacuoles by connection to the extracellular medium in ssTEMs or by localisation to a cell protrusion. Cells in rosette colonies are numbered in order of their appearance along the image stack z-axis. Rosette colony diameters were calculated by measuring the largest distance of the z-axis midsection. Bridge length was measured in one dimension along the bridge midsection. Mean vesicle diameters were calculated from 20 measurements (or as many as possible if the vesicle type was rare) from single cells.

### Data analysis

Univariate differences in the volume and number of subcellular structures between the two cell types were evaluated using two-sample *t* tests. Shapiro–Wilk and Levene’s tests were used to assess normality and homogeneity of variance, respectively. Statistical comparisons were conducted using data scaled against total cell volume. Correlations between colony cell number, cell volume, and bridges per cell were assessed using Pearson correlation tests. All statistical analyses were conducted using R v 3.3.1 [74] implemented in RStudio v 0.99.903 [75].

## Supporting information

S1 Fig **High-magnification TEM panel of the *S*. *rosetta* (A–L) and *O*. *carmela* (M–T) subcellular components discussed herein.** (A) *S*. *rosetta* nucleus showing endoplasmic reticulum, euchromatin, heterochromatin, nuclear membrane, nuclear pore complex and nucleolus. (B) Mitochondrion showing flattened, nondiscoidal cristae. (C) Apical pole showing flagellum, flagellar basal body, nonflagellar basal body, tubulin filaments, and transversal plate. (D) Area of high glycogen storage. (E) Food vacuole. (F) Posterior filopodia projecting from the basal plasma membrane. (G) Golgi apparatus. (H) Microvillus from the apical collar displaying actin filaments. (I–I”) Golgi-associated, electron dense vesicles. (J–J”) Apical, electron-lucent vesicles. (K–K”) Large, extremely electron-lucent vesicles. (L–L”) Extracellular vesicles were observed in two of the single cells and appeared to bud from the microvillar membrane. (M) *O*. *carmela* nucleus showing euchromatin, heterochromatin and nuclear pore complex. (N) Mitochondria displaying cristae. Also visible are cell–cell contacts between two adjacent choanocytes. (O) Collar microvillus. (P) Apical pole and Golgi apparatus showing flagellum, flagellar basal body, nonflagellar basal body, tubulin filaments, and basal foot. (Q) Food vacuole. (R) Rough and smooth endoplasmic reticulum. (S) Basal pole of *O*. *carmela* shows bacteria located in the mesohyl, basal pseudopodia, and endocytotic invagination. (T) Vesicles type 1 (V1) and type 2 (V2) are located throughout the choanocyte cytoplasm. Scale bars = 200 nm, except (L–L”) = 500 nm. af, actin filaments; b, bacteria; bf, basal foot; cc, choanocytes; cr, cristae; dv, food vacuole; er, endoplasmic reticulum; eu, euchromatin; ev, endocytotic invagination; f, flagellum; fbb, flagellar basal body; fp, posterior filopodia; ga, golgi apparatus; gly, glycogen storage; he, heterochromatin; m, mitochondrion; mv, microvillus; n, nucleolus; nfbb, nonflagellar basal body; nm, nuclear membrane; npc, nuclear pore complex; pm, plasma membrane; ps, pseudopodia; rer, rough endoplasmic reticulum; ser, smooth endoplasmic reticulum; TEM, transmission electron microscopy; tf, tubulin filaments; tp, transversal plate
(PDF)

S2 Fig 3D ssTEM reconstructions of high-resolution single and colonial *S*. *rosetta* cells. (A) Gross external morphologies of reconstructions of both single (S1–3) and colonial (C1–3) *S*. *rosetta* cells. (B–C) Structomic reconstructions of single (B) and colonial (C) *S*. *rosetta* cells, with the plasma membrane removed to reveal subcellular ultrastructure. Colours are as in Fig 1. Asterisks indicate engulfed prey bacteria. Cells are labelled with their corresponding cell ID number and volumetric breakdown for each cell is shown below reconstructions. Scale bar = approximately 1 μm. ssTEM, serial ultrathin transmission electron microscopy.
(PDF)

S3 Fig Methodological overview of 3D ssTEM reconstruction of *S*. *rosetta* and *O*. *carmela* cells. (A) ssTEM stacks are imported into the Fiji plugin TrakEM2, aligned, and scaled. Subcellular structures are then manually segmented. (B) 3D ssTEM reconstructions are conducted in TrakEM2 by merging traced structures along the z-axis, initially smoothed and imported into Blender (C). In Blender, final reconstruction artefacts are smoothed using the F Smooth Sculpt Tool and final materials are added for the ultimate render (D). (E) The aforementioned methodology applied to single cells (S1–3), colonial cells (C1–3), a complete RC and a section of an *O*. *carmela* choanocyte chamber. RC, rosette colony; ssTEM, serial ultrathin transmission electron microscopy.
(PDF)

S4 Fig Mean cell volume per colony cell number, intercellular bridges per colony cell number and bridge length. (A) No correlation was found between cell volume and colony cell number. (B) A positive correlation was found between bridges per cell and colony cell number (*P* < 0.05). (C) No apparent pattern was observed between the length of an intercellular bridge and its position along the colony z-axis.
(PDF)

S5 Fig 3D reconstructions and volumetric breakdown of five sponge choanocytes. (A–B) 3D ssTEM reconstructions of five *O*. *carmela* choanocytes and their volumetric breakdown is shown below. Scale bar = approximately 1 μm. ssTEM, serial ultrathin transmission electron microscopy.
(PDF)

S6 Fig Volumetric and numerical comparison of choanocyte and choanoflagellate major subcellular structures. (A) Choanocytes from *O*. *carmela* are significantly larger by volume (μm^3^) than the single and colonial choanoflagellate *S*. *rosetta* cells. Volumetric (%) (±SEM) (nucleus, nucleolus, mitochondria, ER, food vacuoles, and glycogen storage) and numerical (μm^−3^) (±SEM) (mitochondria) differences were found between sponge choanocytes (*n* = 5) and single (*n* = 3) and colonial (*n* = 3) choanoflagellates. *P < 0.05, **P < 0.01, ***P < 0.001. (B–G) TEM and 3D ssTEM reconstructions of amoeboid cell behaviour in sponge choanocytes. Shown are the highly inv and ps basal pole of the choanocyte (B, C), macropinocytotic activity (*) at the apical pole (D, E) and a mesohyl-associated bacterium being engulfed by a ps at the basal pole (F, G). ER, endoplasmatic reticulum; inv, invaginated; ps, pseudopodiated; ssTEM, serial ultrathin transmission electron microscopy.
(PDF)

S1 Movie 3D cellular architecture of choanoflagellate single cell S1. Colours coded as in Fig 1.
(MP4)

S2 Movie 3D cellular architecture of choanoflagellate single cell S2. Colours coded as in Fig 1.
(MP4)

S3 Movie 3D cellular architecture of choanoflagellate single cell S3. Colours coded as in Fig 1.
(MP4)

S4 Movie 3D cellular architecture of choanoflagellate colonial cell C1. Colours coded as in Fig 1.
(MP4)

S5 Movie 3D cellular architecture of choanoflagellate colonial cell C2. Colours coded as in Fig 1.
(MP4)

S6 Movie 3D cellular architecture of choanoflagellate colonial cell C3. Colours coded as in Fig 1.
(MP4)

S7 Movie 3D cellular architecture of choanoflagellate colony RC1. Colours coded as in Fig 3.
(MP4)

S8 Movie 3D cellular architecture of the ‘carrot cell’ from choanoflagellate colony RC3. Colours coded for ‘carrot cell’ as in Fig 3; for all other cells, only the cell bodies are shown.
(MP4)

S9 Movie 3D cellular architecture of the ‘chili cell’ from choanoflagellate colony RC4. Colours coded for ‘chili cell’ as in Fig 3; for all other cells, only the cell bodies are shown.
(MP4)

S10 Movie 3D cellular architecture of sponge choanocytes. Colours coded as in Fig 4.
(M4V)

S11 Movie 3D reconstruction of choanoflagellate apical pole. Colours coded as in Fig 4.
(MP4)

S12 Movie 3D reconstruction of sponge choanocyte apical pole. Colours coded as in Fig 4.
(MP4)

S1 Table Volumetric measurements of *S*. *rosetta* cells and components. (DOCX)

S2 Table Numbers of various organelles and components in *S*. *rosetta* cells. (DOCX)

S3 Table Volumetric measurements of *O*. *carmela* choanocytes and components. (DOCX)

S4 Table Numbers of various organelles and components in *O*. *carmela* choanocytes. (DOCX)

S1 Data (XLSX)

## Abbreviations

ECM: extracellular matrix
ER: endoplasmic reticulum
RC: rosette colony
RIF: rosette-inducing factor
SNARE: soluble N-ethylmaleimide-sensitive-factor attachment receptor
ssTEM: serial ultrathin transmission electron microscopy
