# Supplementary figures and images for: The architecture of cell differentiation in choanoflagellates and sponge choanocytes
Source: PLoS Biol. 2019 Apr 12;17(4):e3000226. doi: 10.1371/journal.pbio.3000226 (PMC6481868; doi:10.1371/journal.pbio.3000226)

Suppl. Figure 1

Choanoflagellate

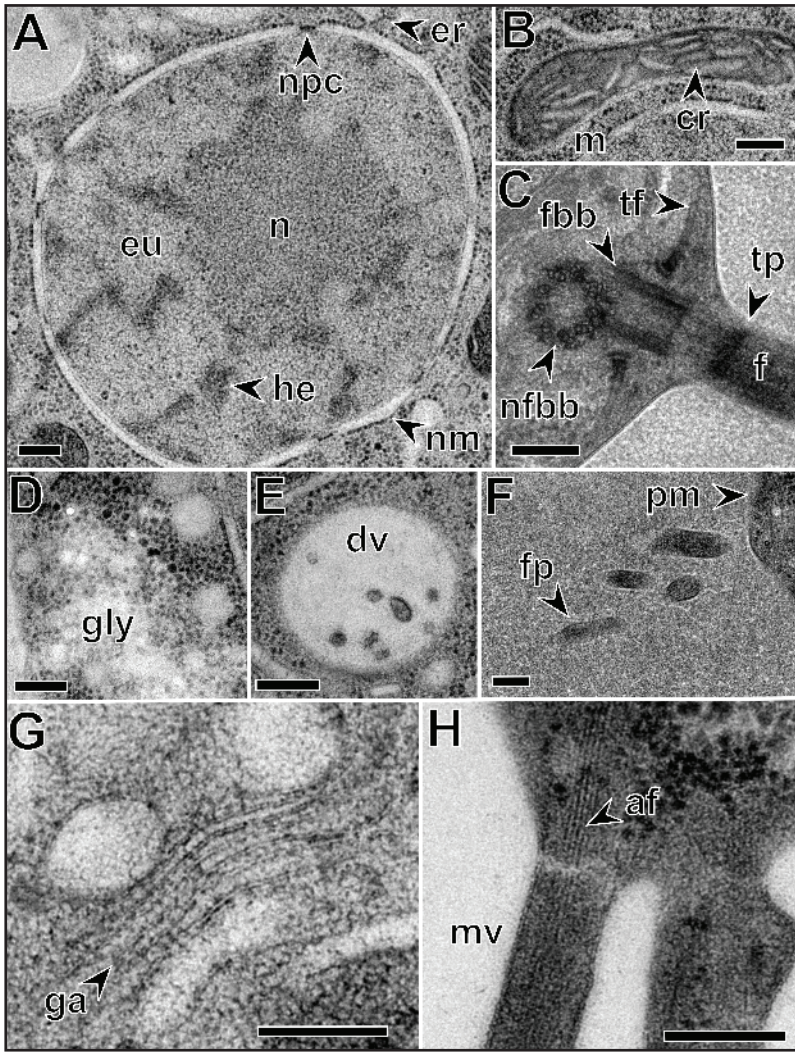

Sponge

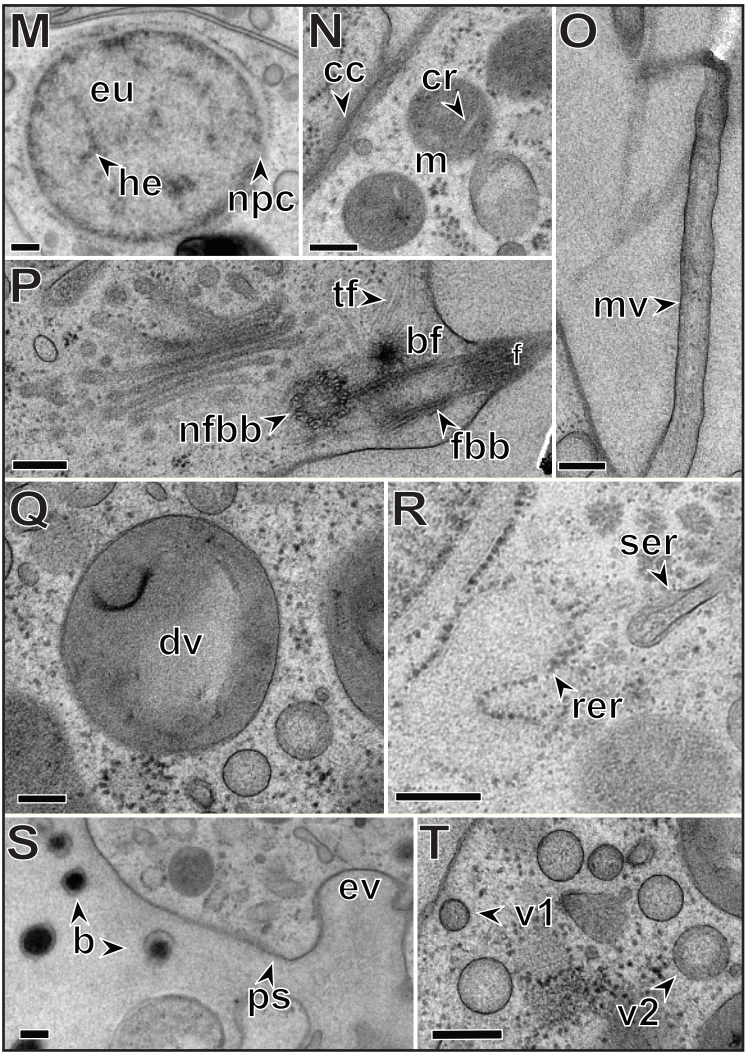

Choanoflagellate vesicles

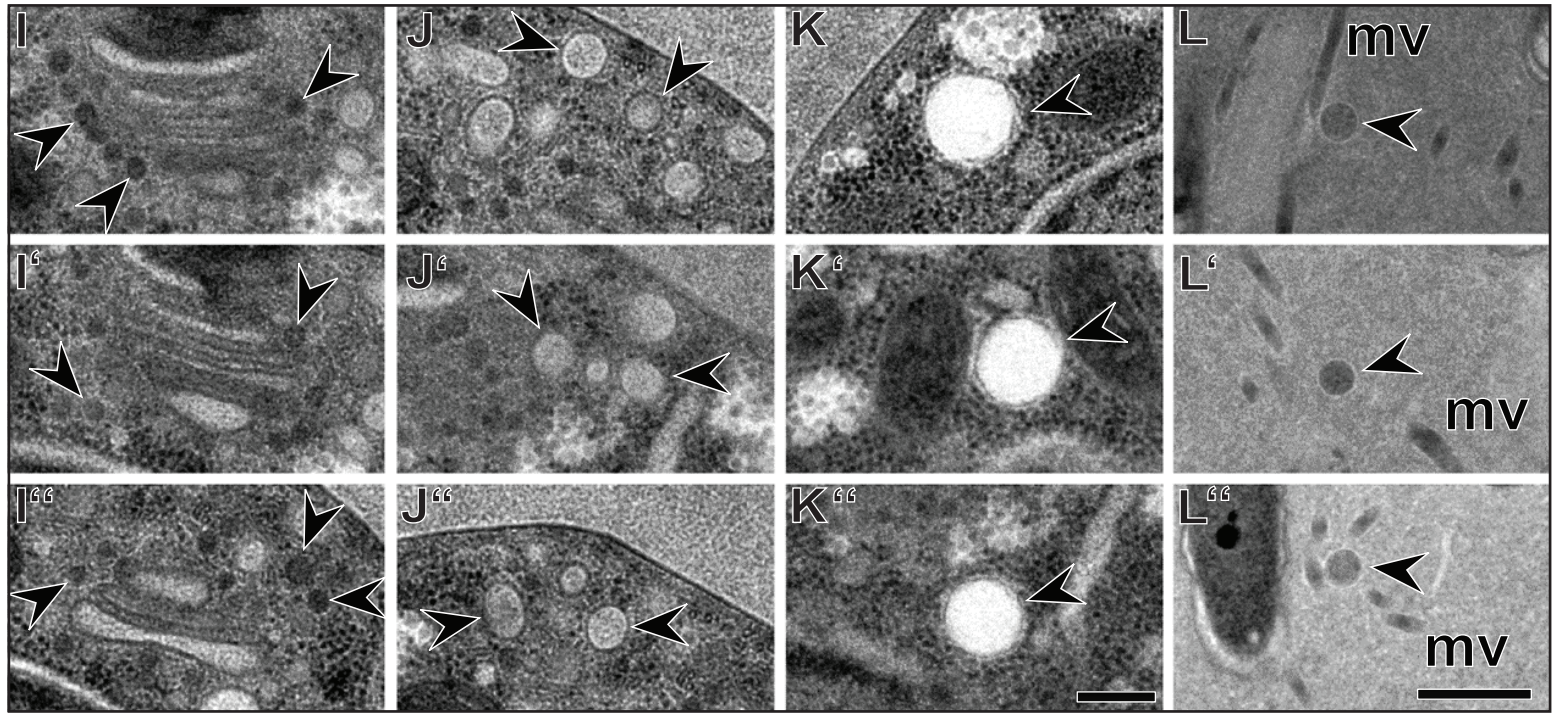

Supplement: S1 Fig — High-magnification TEM panel of the S. rosetta (A–L) and O. carmela (M–T) subcellular components discussed herein. (A) S. rosetta nucleus showing endoplasmic reticulum, euchromatin, heterochromatin, nuclear membrane, nuclear pore complex and nucleolus. (B) Mitochondrion showing flattened, nondiscoidal cristae. (C) Apical pole showing flagellum, flagellar basal body, nonflagellar basal body, tubulin filaments, and transversal plate. (D) Area of high glycogen storage. (E) Food vacuole. (F) Posterior filopodia projecting from the basal plasma membrane. (G) Golgi apparatus. (H) Microvillus from the apical collar displaying actin filaments. (I–I”) Golgi-associated, electron dense vesicles. (J–J”) Apical, electron-lucent vesicles. (K–K”) Large, extremely electron-lucent vesicles. (L–L”) Extracellular vesicles were observed in two of the single cells and appeared to bud from the microvillar membrane. (M) O. carmela nucleus showing euchromatin, heterochromatin and nuclear pore complex. (N) Mitochondria displaying cristae. Also visible are cell–cell contacts between two adjacent choanocytes. (O) Collar microvillus. (P) Apical pole and Golgi apparatus showing flagellum, flagellar basal body, nonflagellar basal body, tubulin filaments, and basal foot. (Q) Food vacuole. (R) Rough and smooth endoplasmic reticulum. (S) Basal pole of O. carmela shows bacteria located in the mesohyl, basal pseudopodia, and endocytotic invagination. (T) Vesicles type 1 (V1) and type 2 (V2) are located throughout the choanocyte cytoplasm. Scale bars = 200 nm, except (L–L”) = 500 nm. af, actin filaments; b, bacteria; bf, basal foot; cc, choanocytes; cr, cristae; dv, food vacuole; er, endoplasmic reticulum; eu, euchromatin; ev, endocytotic invagination; f, flagellum; fbb, flagellar basal body; fp, posterior filopodia; ga, golgi apparatus; gly, glycogen storage; he, heterochromatin; m, mitochondrion; mv, microvillus; n, nucleolus; nfbb, nonflagellar basal body; nm, nuclear membrane; npc, nuclear pore co [file pbio.3000226.s001.pdf]

Suppl. Figure 2

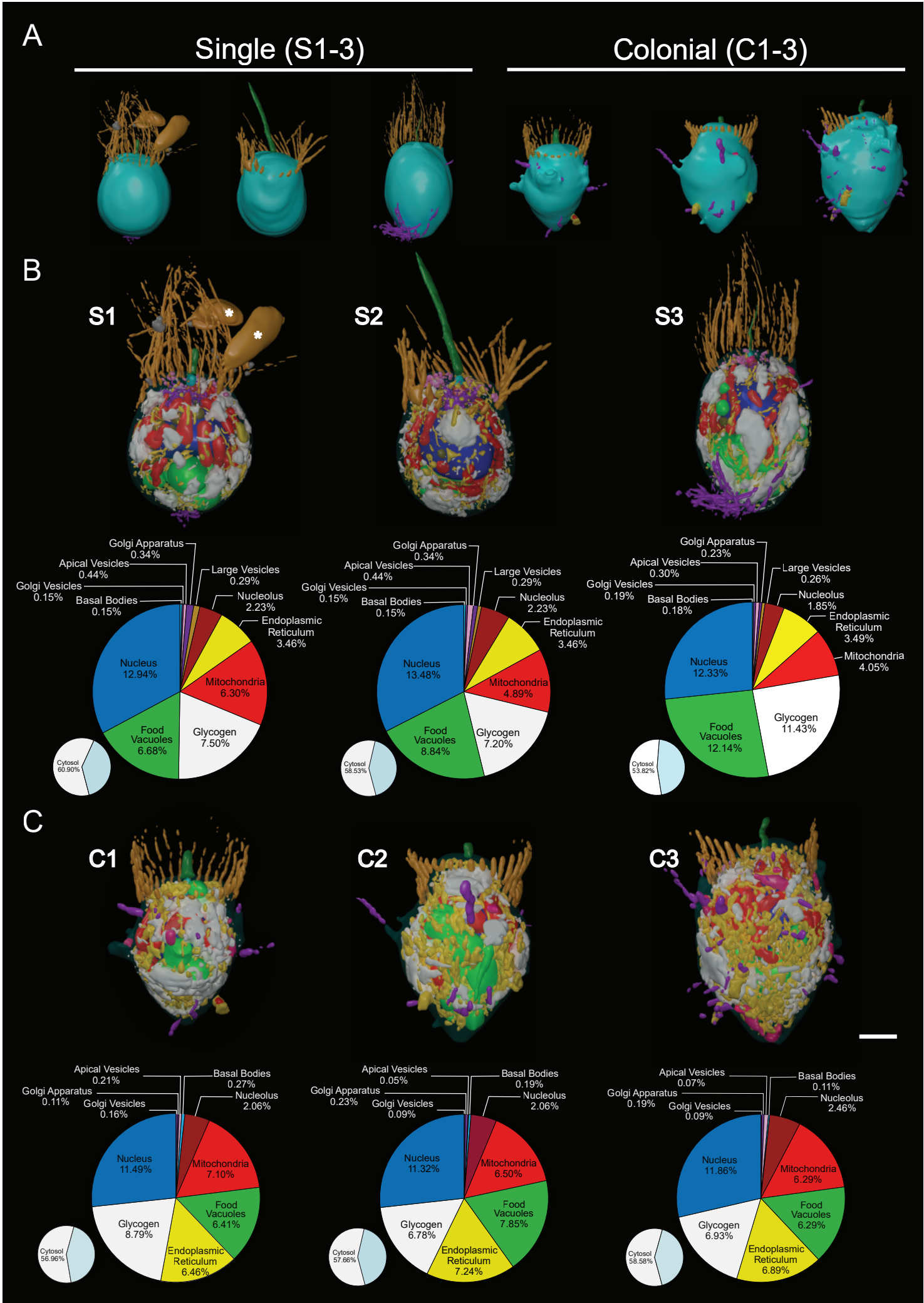

Supplement: S2 Fig — (A) Gross external morphologies of reconstructions of both single (S1–3) and colonial (C1–3) S. rosetta cells. (B–C) Structomic reconstructions of single (B) and colonial (C) S. rosetta cells, with the plasma membrane removed to reveal subcellular ultrastructure. Colours are as in Fig 1. Asterisks indicate engulfed prey bacteria. Cells are labelled with their corresponding cell ID number and volumetric breakdown for each cell is shown below reconstructions. Scale bar = approximately 1 μm. ssTEM, serial ultrathin transmission electron microscopy. (PDF) [file pbio.3000226.s002.pdf]

Suppl. Figure 3

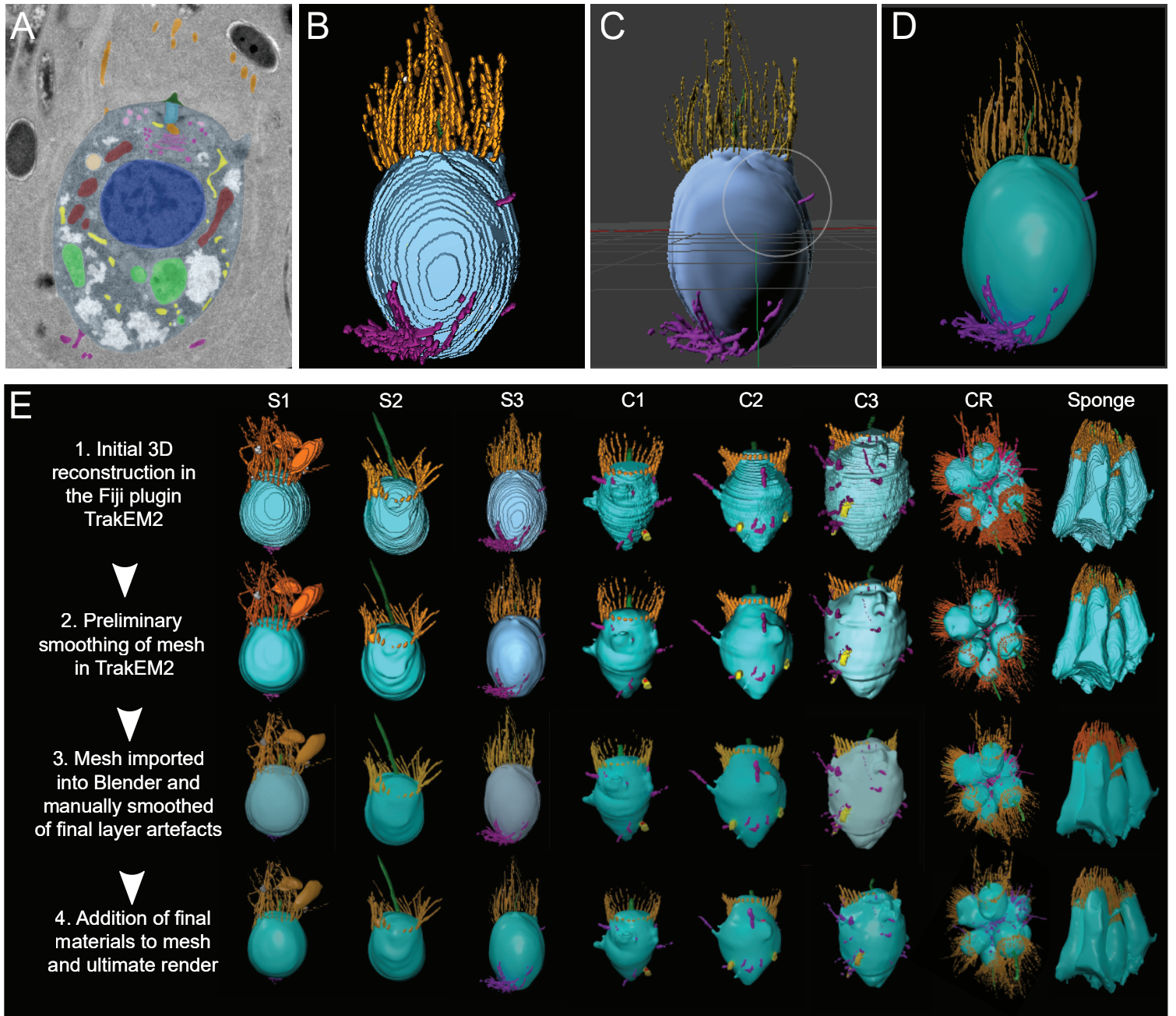

Supplement: S3 Fig — (A) ssTEM stacks are imported into the Fiji plugin TrakEM2, aligned, and scaled. Subcellular structures are then manually segmented. (B) 3D ssTEM reconstructions are conducted in TrakEM2 by merging traced structures along the z-axis, initially smoothed and imported into Blender (C). In Blender, final reconstruction artefacts are smoothed using the F Smooth Sculpt Tool and final materials are added for the ultimate render (D). (E) The aforementioned methodology applied to single cells (S1–3), colonial cells (C1–3), a complete RC and a section of an O. carmela choanocyte chamber. RC, rosette colony; ssTEM, serial ultrathin transmission electron microscopy. (PDF) [file pbio.3000226.s003.pdf]

Suppl. Figure 4

A

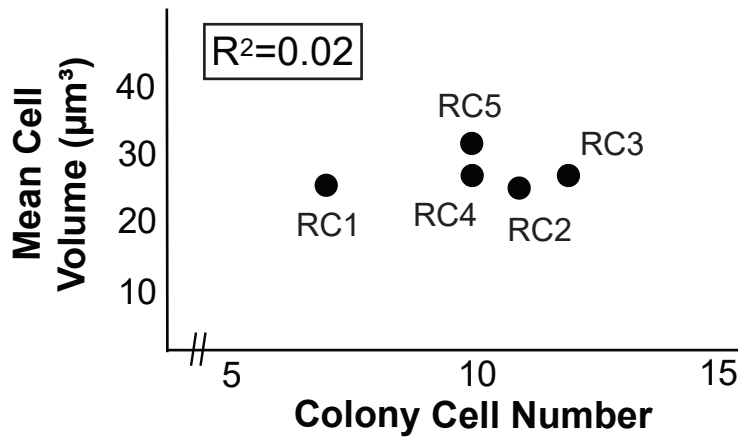

B

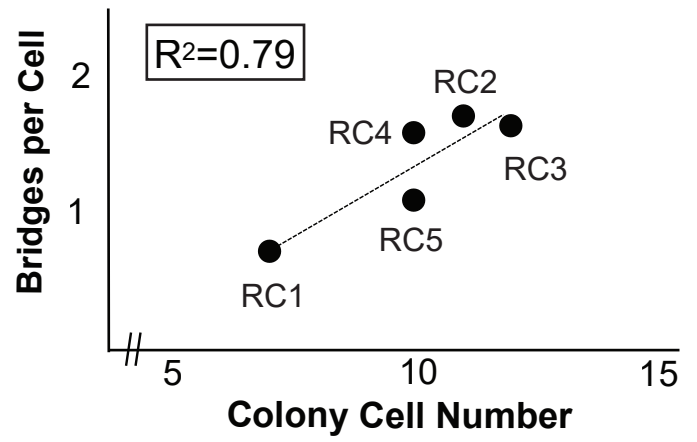

C

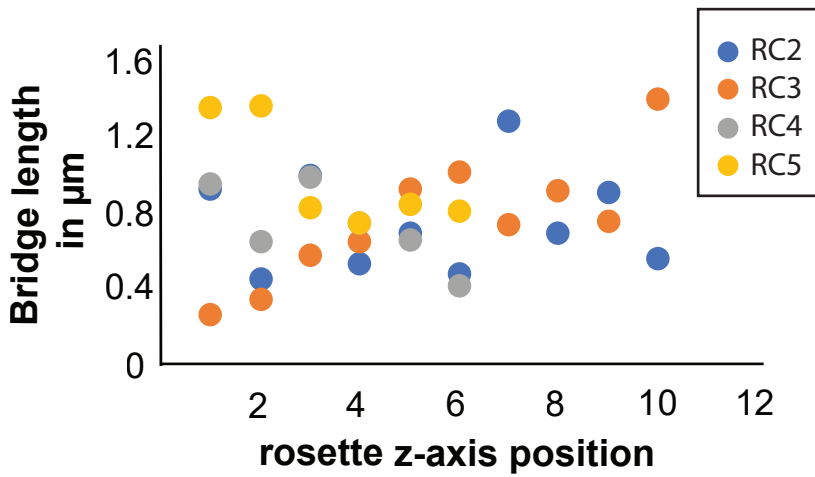

Supplement: S4 Fig — (A) No correlation was found between cell volume and colony cell number. (B) A positive correlation was found between bridges per cell and colony cell number (P < 0.05). (C) No apparent pattern was observed between the length of an intercellular bridge and its position along the colony z-axis. (PDF) [file pbio.3000226.s004.pdf]

Suppl. Figure 5

A

Cell 1

Cell 2

Cell 3

Cell 4

Cell 5

B

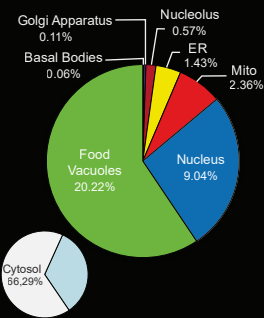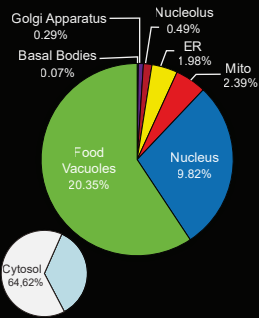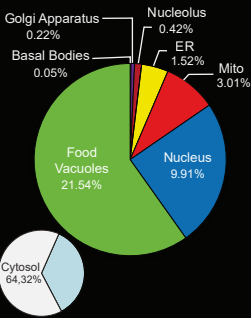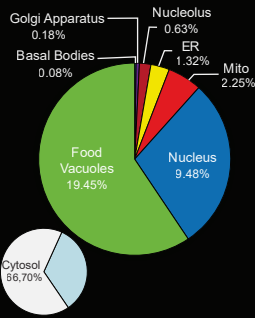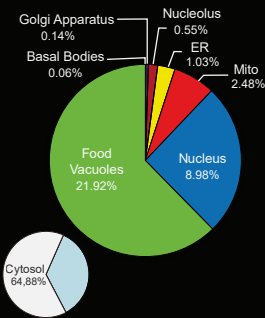

Supplement: S5 Fig — (A–B) 3D ssTEM reconstructions of five O. carmela choanocytes and their volumetric breakdown is shown below. Scale bar = approximately 1 μm. ssTEM, serial ultrathin transmission electron microscopy. (PDF) [file pbio.3000226.s005.pdf]

Suppl. Figure 6

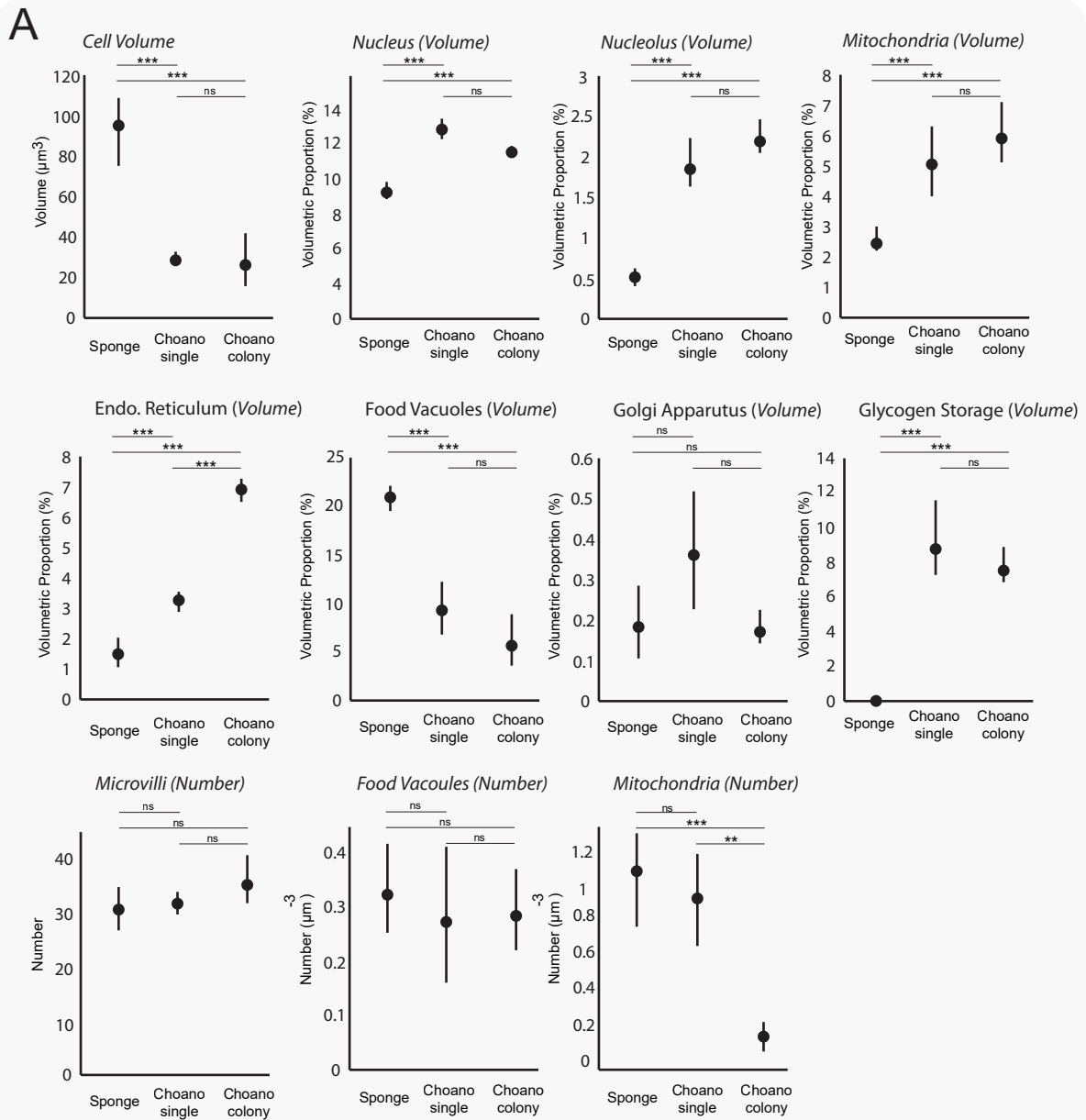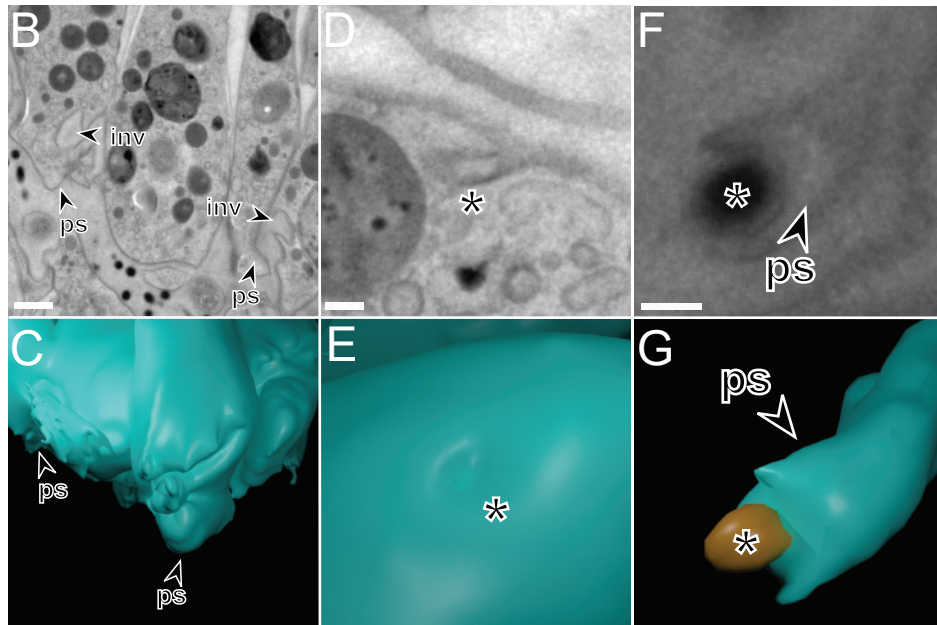

Supplement: S6 Fig — (A) Choanocytes from O. carmela are significantly larger by volume (μm3) than the single and colonial choanoflagellate S. rosetta cells. Volumetric (%) (±SEM) (nucleus, nucleolus, mitochondria, ER, food vacuoles, and glycogen storage) and numerical (μm−3) (±SEM) (mitochondria) differences were found between sponge choanocytes (n = 5) and single (n = 3) and colonial (n = 3) choanoflagellates. *P < 0.05, **P < 0.01, ***P < 0.001. (B–G) TEM and 3D ssTEM reconstructions of amoeboid cell behaviour in sponge choanocytes. Shown are the highly inv and ps basal pole of the choanocyte (B, C), macropinocytotic activity (*) at the apical pole (D, E) and a mesohyl-associated bacterium being engulfed by a ps at the basal pole (F, G). ER, endoplasmatic reticulum; inv, invaginated; ps, pseudopodiated; ssTEM, serial ultrathin transmission electron microscopy. (PDF) [file pbio.3000226.s006.pdf]
