# Supplementary material for: The architecture of cell differentiation in choanoflagellates and sponge choanocytes
Source: PLoS Biol. 2019 Apr 12;17(4):e3000226. doi: 10.1371/journal.pbio.3000226 (PMC6481868; doi:10.1371/journal.pbio.3000226)
Supplement: S1 Table — (DOCX) [file pbio.3000226.s019.docx]

**Table S1. Volumetric measurements of *S. rosetta* cells and components**

|  | **Single cells** | | | | **Colonial cells** | | | |
| --- | --- | --- | --- | --- | --- | --- | --- | --- |
| **Organelle** | **S1** | **S2** | **S3** | **Mean +/- SD** | **C1** | **C2** | **C3** | **Mean +/- SD** |
| Cell Body | 26.81 (100) | 32.34  (100) | 26.68  (100) | 28.61 ± 3.23  (100 ± 0) | 18.89  (100) | 21.54  (100) | 41.82  (100) | 27.41 ± 12.54  (100 ± 0) |
| Nucleus | 3.92  (12.94) | 5.08  (13.48) | 3.73  (12.33) | 4.24 ± 0.73  (12.92 ± 0.58) | 2.56  (11.49) | 2.89  (11.33) | 5.99  (11.86) | 3.81 ± 1.89  (11.56 ± 0.27) |
| Nucleolus | 0.45  (1.68) | 0.72  (2.23) | 0.44  (1.65) | 0.54 ± 0.16  (1.85 ± 0.33) | 0.39  (2.06) | 0.45  (2.09) | 1.03  (2.46) | 0.62 ± 0.35  (2.2 ± 0.22) |
| Mitochondria | 1.69  (6.30) | 1.58  (4.89) | 1.08  (4.05) | 1.45 ± 0.33  (5.08 ± 1.14) | 1.34  (7.10) | 1.4  (6.50) | 2.63  (6.29) | 1.79 ± 0.73  (6.63 ± 0.42) |
| Endoplasmic Reticulum | 0.77  (2.87) | 1.12  (3.46) | 0.93  (3.49) | 0.94 ± 0.18  (3.27 ± 0.35) | 1.22  (6.46) | 1.56  (7.24) | 2.88  (6.89) | 1.89 ± 0.88  (6.86 ± 0.39) |
| Food Vacuoles | 1.79  (6.68) | 2.86  (8.84) | 3.24  (12.14) | 2.63 ± 0.75  (9.22 ± 2.75) | 1.21  (6.41) | 1.69  (7.85) | 2.63  (6.29) | 1.84 ± 0.72  (6.85 ± 0.87) |
| Glycogen Storage | 2.01  (7.50) | 2.33  (7.20) | 3.05  (11.43) | 2.46 ± 0.53  (8.71 ± 2.36) | 1.66  (8.79) | 1.46  (6.78) | 2.9  (6.93) | 2.01 ± 0.78  (7.50 ± 1.12) |
| Flagellar Basal Body | 0.02  (0.07) | 0.03  (0.09) | 0.01  (0.10) | 0.02 ± 0.01  (0.09 ± 0.02) | 0.03  (0.16) | 0.03  (0.14) | 0.02  (0.05) | 0.03 ± 0.01  (0.12 ± 0.06) |
| Non-Flagellar Basal Body | 0.02  (0.07) | 0.02  (0.06) | 0.02  (0.08) | 0.02 ± 0  (0.07 ± 0.01) | 0.02  (0.11) | 0.01  (0.05) | 0.02  (0.05) | 0.02 ± 0.01  (0.07 ± 0.03) |
| Golgi Apparatus | 0.14  (0.52) | 0.11  (0.34) | 0.06  (0.23) | 0.10 ± 0.04  (0.36 ± 0.15) | 0.02  (0.11) | 0.05  (0.23) | 0.08  (0.19) | 0.05 ± 0.03  (0.18 ± 0.06) |
| Golgi Associated Vesicles | 0.03  (0.12) | 0.05  (0.15) | 0.05  (0.19) | 0.04 ± 0.01  (0.15 ± 0.04) | 0.03  (0.16) | 0.02  (0.09) | 0.03  (0.07) | 0.03 ± 0.01  (0.11 ± 0.05) |
| Apical Vesicles | 0.06  (0.22) | 0.14  (0.44) | 0.08  (0.30) | 0.09 ± 0.04  (0.32 ± 0.11) | 0.04  (0.21) | 0.01  (0.05) | 0.14  (0.33) | 0.06 ± 0.07  (0.20 ± 0.14) |
| Large Vesicles | 0.03  (0.45) | 0.09  (0.29) | 0.07  (0.26) | 0.06 ± 0.03  (0.33 ± 0.10) | 0  (0) | 0  (0) | 0  (0) | 0  (0) |
| Extracellular Vesicles | 0.06  (0.22) | 0  (0) | 0.01  (0.04) | 0.02 ± 0.03  (0.09 ± 0.12) | 0  (0) | 0  (0) | 0  (0) | 0  (0) |
| Endocytotic Vacuoles | 0.04  (0.15) | 0  (0) | 0.02  (0.07) | 0.02 ± 0.02  (0.07 ± 0.07) | 0.06  (0.32) | 0.04  (0.19) | 0.18  (0.43) | 0.09 ± 0.08  (0.32 ± 0.12) |
| Filopodia | 0.07  (0.26) | 0  (0) | 0.28  (1.05) | 0.12 ± 0.15  (0.44 ± 0.55) | 0.07  (0.37) | 0.08  (0.37) | 0.18  (0.43) | 0.11 ± 0.06  (0.39 ± 0.03) |
| Cytosol | 16.33  (60.9) | 18.93  (58.53) | 14.36  (53.82) | 16.54 ± 2.29  (57.75 ± 3.6) | 10.76  (56.96) | 12.42  (57.66) | 24.50  (58.58) | 15.89 ± 7.49  (57.97 ± 0.81) |

Volumes were measured in μm^3^.

Values between parentheses are percentages of cell volume.
