# Supplementary material for: The architecture of cell differentiation in choanoflagellates and sponge choanocytes
Source: PLoS Biol. 2019 Apr 12;17(4):e3000226. doi: 10.1371/journal.pbio.3000226 (PMC6481868; doi:10.1371/journal.pbio.3000226)
Supplement: S2 Table — (DOCX) [file pbio.3000226.s020.docx]

**Table S2. Numbers of various organelles and components in *S. rosetta* cells**

|  | **Single cells** | | | | **Colonial cells** | | | |
| --- | --- | --- | --- | --- | --- | --- | --- | --- |
| **Organelle** | **S1** | **S2** | **S3** | **Mean +/- SD** | **C1** | **C2** | **C3** | **Mean +/- SD** |
| Nucleus | 1 | 1 | 1 | 1 ± 0 | 1 | 1 | 1 | 1 ± 0 |
| Nucleolus | 1 | 1 | 1 | 1 ± 0 | 1 | 1 | 1 | 1 ± 0 |
| Flagellum | 1 | 1 | 1 | 1 ± 0 | 1 | 1 | 1 | 1 ± 0 |
| Flagellar Basal Body | 1 | 1 | 1 | 1 ± 0 | 1 | 1 | 1 | 1 ± 0 |
| Non-Flagellar Basal Body | 1 | 1 | 1 | 1 ± 0 | 1 | 1 | 1 | 1 ± 0 |
| Microvilli | 30 | 34 | 32 | 32 ± 2 | 32 | 33 | 41 | 35.3 ± 4.9 |
| Golgi Apparatus | 1 | 1 | 1 | 1 ± 0 | 1 | 1 | 1 | 1 ± 0 |
| Golgi Associated Vesicles | 140 | 203 | 156 | 166.3 ± 32.7 | 64 | 51 | 102 | 72.3 ± 26.5 |
| Food Vacuoles | 6 | 8 | 11 | 8.3 ± 2.5 | 5 | 9 | 9 | 7.7 ± 2.3 |
| Mitochondria | 32 | 22 | 22 | 25.3 ± 5.8 | 1 | 3 | 9 | 4.3 ± 4.2 |
| Apical Vesicles | 41 | 53 | 68 | 54 ± 13.5 | 23 | 8 | 64 | 31.6 ± 29.0 |
| Large Vesicles | 11 | 12 | 7 | 10 ± 2.7 | 0 | 0 | 0 | 0 |
| Extracellular Vesicles | 12 | 0 | 4 | 5.3 ± 6.1 | 0 | 0 | 0 | 0 |
| Endocytotic Vacuoles | 1 | 0 | 2 | 1 ± 1 | 5 | 3 | 7 | 5 ± 2 |
| Pseudopodia | 0 | 1 | 2 | 1 ± 1 | 6 | 8 | 10 | 8 ± 2 |
| Intercellular Bridges | 0 | 0 | 0 | 0 | 2 | 2 | 2 | 2 ± 0 |
