# Supplementary material for: The architecture of cell differentiation in choanoflagellates and sponge choanocytes
Source: PLoS Biol. 2019 Apr 12;17(4):e3000226. doi: 10.1371/journal.pbio.3000226 (PMC6481868; doi:10.1371/journal.pbio.3000226)
Supplement: S3 Table — (DOCX) [file pbio.3000226.s021.docx]

**Table S3. Volumetric measurements of *O. carmela* choanocytes and components**

|  | **Five sponge choanocytes** | | | | | |
| --- | --- | --- | --- | --- | --- | --- |
| **Organelle** | **Cell 1** | **Cell 2** | **Cell 3** | **Cell 4** | **Cell 5** | **Mean +/- SD** |
| Cell Body | 108.56  (100) | 75.35  (100) | 107.37  (100) | 83.95  (100) | 100.29  (100) | 95.1 ± 14.8  (100 ± 0) |
| Nucleus | 10.47  (9.07) | 7.77  (9.82) | 10.02  (8.91) | 8.49  (9.48) | 9.54  (8.98) | 9.26 ± 1.11  (9.25 ± 0.39) |
| Nucleolus | 0.62  (0.57) | 0.37  (0.49) | 0.45  (0.42) | 0.53  (0.63) | 0.55  (0.55) | 0.51 ± 0.10  (0.53 ± 0.08) |
| Mitochondria | 2.56  (2.36) | 1.8  (2.39) | 3.23  (3.01) | 1.89  (2.25) | 2.49  (2.48) | 2.39 ± 0.58  (2.50 ± 0.30) |
| Endoplasmic Reticulum | 1.43  (1.32) | 1.49  (1.98) | 1.63  (1.52) | 1.03  (1. 32) | 1.03  (1.03) | 1.32 ± 0.28  (1.43 ± 0.35) |
| Food Vacuoles | 21.95  (20.22) | 15.33  (20.35) | 23.13  (21.54) | 16.33  (19.45) | 21.98  (21.92) | 19.74 ± 3.62  (20.70 ± 1.01) |
| Glycogen Storage | 0  (0) | 0  (0) | 0  (0) | 0  (0) | 0  (0) | 0  (0) |
| Flagellar Basal Body | 0.03  (0.03) | 0.02  (0.03) | 0.03  (0.03) | 0.03  (0.04) | 0.02  (0.02) | 0.03 ± 0.01  (0.03 ± 0.02) |
| Non-Flagellar Basal Body | 0.03  (0.03) | 0.03  (0.04) | 0.02  (0.02) | 0.03  (0.04) | 0.02  (0.02) | 0.03 ± 0.01  (0.03 ± 0.01) |
| Golgi Apparatus | 0.12  (0.11) | 0.22  (0.29) | 0.24  (0.22) | 0.15  (0.18) | 0.14  (0.14) | 0.17 ± 0.05  (0.19 ± 0.07) |
| Filopodia | 0  (0) | 0  (0) | 0  (0) | 0  (0) | 0  (0) | 0  (0) |
| Cytosol | 71.97  (66.29) | 48.69  (64.62) | 69.07  (64.32) | 56  (66.70) | 65.07  (64.88) | 62.16 ± 9.64  (65.36 ± 1.06) |

Volumes were measured in μm^3^.

Values between parentheses are percentages of cell volume.
