# Supplementary material for: The architecture of cell differentiation in choanoflagellates and sponge choanocytes
Source: PLoS Biol. 2019 Apr 12;17(4):e3000226. doi: 10.1371/journal.pbio.3000226 (PMC6481868; doi:10.1371/journal.pbio.3000226)
Supplement: S4 Table — (DOCX) [file pbio.3000226.s022.docx]

**Table S4. Numbers of various organelles and components in *O. carmela* choanocytes**

|  | **Five sponge choanocytes** | | | | | |
| --- | --- | --- | --- | --- | --- | --- |
| **Organelle** | **Cell 1** | **Cell 2** | **Cell 3** | **Cell 4** | **Cell 5** | **Mean +/- SD** |
| Nucleus | 1 | 1 | 1 | 1 | 1 | 1 ± 0 |
| Nucleolus | 1 | 1 | 1 | 1 | 1 | 1 ± 0 |
| Flagellum | 1 | 1 | 1 | 1 | 1 | 1 ± 0 |
| Flagellar Basal Body | 1 | 1 | 1 | 1 | 1 | 1 ± 0 |
| Non-Flagellar Basal Body | 1 | 1 | 1 | 1 | 1 | 1 ± 0 |
| Microvilli | 35 | 27 | 35 | 27 | 29 | 30.6 ± 4.1 |
| Golgi Apparatus | 1 | 1 | 1 | 1 | 1 | 1 ± 0 |
| Food Vacuoles | 42 | 22 | 27 | 35 | 27 | 30.6 ± 7.9 |
| Mitochondria | 125 | 81 | 140 | 66 | 117 | 105.8 ± 31.1 |
| Pseudopodia | 0 | 0 | 0 | 0 | 0 | 0 |
| Intercellular Bridges | 0 | 0 | 0 | 0 | 0 | 0 |
